# Identification of *Arabidopsis thaliana* small RNAs responsive to the fungal pathogen *Botrytis cinerea* at an early stage of interaction

**DOI:** 10.1371/journal.pone.0304790

**Published:** 2024-06-14

**Authors:** Emir Alejandro Padilla-Padilla, Carlos De la Rosa, Wendy Aragón, Ana Karen Ávila-Sandoval, Martha Torres, Ana Elena Dorantes-Acosta, Mario A. Arteaga-Vázquez, Damien Formey, Mario Serrano

**Affiliations:** 1 Centro de Ciencias Genómicas, Universidad Nacional Autónoma de México, Cuernavaca, Morelos, México; 2 Posgrado en Ciencias Biológicas, Unidad de Posgrado, Ciudad Universitaria, Coyoacán, Ciudad de México; 3 Instituto de Biociencias, Universidad Autónoma de Chiapas, Chiapas, México; 4 Instituto de Biotecnología y Ecología Aplicada (INBIOTECA), Universidad Veracruzana, Xalapa, Veracruz, México; Nuclear Science and Technology Research Institute, ISLAMIC REPUBLIC OF IRAN

## Abstract

In plants, small RNAs (sRNAs), mainly microRNAs (miRNAs) and small interfering RNAs (siRNAs), have been described as key regulators of plant development, growth, and abiotic and biotic responses. Despite reports indicating the involvement of certain sRNAs in regulating the interaction between *Botrytis cinerea* (a major necrotrophic fungal phytopathogen) and host plants, there remains a lack of analysis regarding the potential regulatory roles of plant sRNAs during early stages of the interaction despite early immune responses observed then during infection. We present the first transcriptome-wide analysis of small RNA expression on the early interaction between the necrotrophic fungus *Botrytis cinerea* and the model plant *Arabidopsis thaliana*. We found that evolutionary conserved *A*. *thaliana* miRNAs were the sRNAs that accumulated the most in the presence of *B*. *cinerea*. The upregulation of miR167, miR159 and miR319 was of particular interest because these, together with their target transcripts, are involved in the fine regulation of the plant hormone signaling pathways. We also describe that miR173, which triggers the production of secondary siRNAs from TAS1 and TAS2 loci, as well as secondary siRNAs derived from these loci, is upregulated in response to *B*. *cinerea*. Thus, at an early stage of the interaction there are transcriptional changes of sRNA-guided silencing pathway genes and of a subset of sRNAs that targeted genes from the PPR gene superfamily, and these may be important mechanisms regulating the interaction between *A*. *thaliana* and *B*. *cinerea*. This work provides the basis for a better understanding of the regulation mediated by sRNAs during early *B*. *cinerea-*plant interaction and may help in the development of more effective strategies for its control.

## Introduction

*Botrytis cinerea* is a widespread necrotrophic fungus with a broad host range that can infect more than 1,400 plant species, mainly angiosperms [1]. *B*. *cinerea* represents a food safety hazard as many of the affected plants are important crops for human consumption, which highlights the importance to mitigate the numerous losses caused by this pathogen worldwide each year [2,3]. In many of its host plants, *B*. *cinerea* causes rot characterized by the softening of infected tissues, the development of dark lesions and, in more advanced stages of infection, growth of mycelium and sporulation. The use of fungicides is currently the principal means of control of *B*. *cinerea*; however, it has been reported that some populations of this phytopathogen have developed resistance to a variety of fungicide compounds [4]. Understanding the molecular mechanisms that govern the interaction between *B*. *cinerea* and its host plants to design rational control strategies has been pointed out as an alternative to fungicides [5]. *B*. *cinerea* infection on leaves of the model plant *Arabidopsis thaliana* is currently one of the main systems in which the molecular mechanisms regulating the interaction of this fungus with plants are being studied.

The infection of *A*. *thaliana* by *B*. *cinerea* progresses through two main stages. The first stage, the primary lesion, occurs after conidia attachment and germination on the plant surface. This stage is marked by the localized death of plant cells beneath the site of fungal penetration. Following a period of quiescence, the infection enters the later stage, characterized by lesion expansion, maceration of plant tissue and sporulation of *B*. *cinerea* [6,7]. *B*. *cinerea* produces several different molecules (virulence factors) that contribute to the infection process [1,8]. After conidial attachment and germination on the plant surface, during cuticle penetration, enzymes are secreted from *B*. *cinerea* appressoria whose activities promote an oxidative burst in the plant-fungal interphase [9]. *B*. *cinerea* also produces a variety of other enzymes and metabolites that degrade the plant cell wall or act as phytotoxic molecules [5,8]. The plant then also triggers the production of reactive oxygen species (ROS), contributing to the oxidative burst. This accumulation of ROS represents a perturbation of the redox status of the plant, promoting a hypersensitive response (HR), characterized by programmed cell death at the infection site. Plant cell death, either regulated, as in programmed cell death or in a more unsubtle manner through necrosis by the phytotoxic fungal compounds, contributes to the progression of infection [10].

For their part, plants can perceive patterns in molecules such as chitin or oligogalacturonides (cell wall degradation products), which respectively come from the fungus and from the plant itself as a result of the damage caused by the fungus. These molecular patterns, respectively called Microbe Associated Molecular Patterns (MAMPs) and Damage Associated Molecular Patterns (DAMPs), are perceived through receptors and co-receptors (Receptor-Like Kinases (RLKs) and Receptor-Like Proteins (RLPs)) located in the plant cell plasma membrane [5,11]. Once MAMPs and DAMPs are perceived, the signals are internalized into the plant cell through complex signaling networks, involving Receptor-Like Cytoplasmic Kinases (RLCKs) and MAPK-dependent and independent pathways, to activate plant defense responses. These defense responses mainly consist of transcriptional regulation of transcription factors and genes for biosynthesis and perception of plant hormones (including jasmonic acid, ethylene, salicylic acid, auxins and abscisic acid) to modulate the expression of genes devoted to the reinforcement of the plant physical barriers, the biosynthesis of antimicrobial molecules and the modulation of plant cell death responses [5]. There is also substantial evidence for a role of plant endogenous small RNAs (sRNAs) in the regulation of gene expression in immune responses [12–14].

sRNAs are single-stranded RNAs of approximately 20 to 24 nucleotides encoded in the genomes of eukaryotic organisms which negatively regulate target gene expression, at the transcriptional or post-transcriptional level, through mechanisms guided by sequence complementarity between the sRNAs and their targets [15–17]. In plants, sRNAs can be broadly classified into different categories according to their biogenesis mechanisms: sRNAs derived from single-stranded RNA precursors that fold into self-complementary stem-loop structures (hpRNAs) and those derived from precursors formed by two complementary strands of RNA (siRNAs), with some other less characterized categories, such as the one in which the sRNA precursors are tRNAs (tsRNAs) [17–19]. microRNAs (miRNAs), the main class of hpRNAs, are sRNAs processed with precision from their precursors and mostly regulate their targets at the post-transcriptional level. Within siRNAs there are other subclasses such as secondary siRNAs and hc-siRNAs. Secondary siRNAs are sRNAs produced from a primary transcript that undergo a miRNA-guided cleavage with one of the resulting segments being processed into a double-stranded RNA and sliced into consecutive segments of 21 nucleotides starting from the cleavage site, producing the secondary siRNAs. hc-siRNAs are mainly generated from transposons, and repetitive or heterochromatic regions in the plant genome and commonly mediate the repression at the transcriptional level of genomic regions around their production site, regulating genome stability by means of recruiting factors for DNA methylation and chromatin remodeling [17–19]. miRNAs, secondary siRNAs and hc-siRNAs are all processed and generated by Dicer-like (DCL) proteins and regulate gene expression by guiding proteins from the Argonaute (AGO) family [17–19].

Changes in sRNA expression in the presence of various pathogens, as well as changes in pathogen susceptibility in plant mutants in sRNA genes or in sRNA-guided silencing pathway genes, underscore the pivotal roles of sRNAs as key regulators in plant immune responses to a diversity of pathogens [12,14]. More specifically, some sRNAs have turned out to be regulators in the response to *B*. *cinerea*. Analysis of tomato leaves indicated that the upregulation of the miRNA miR319 (probably by its role as a jasmonic acid signaling regulator) and the downregulation of hc-siRNAs are molecular mechanisms related to the induction of immune responses to *B*. *cinerea* [20]. On the other hand, miR394 has been suggested to be a negative regulator of immune responses to this fungus in tomato leaves and in *A*. *thaliana* [21]. Weiberg et al. (2013) and Wang et al. (2017) reported that sRNAs are also part of the virulence factors produced by *B*. *cinerea* when infecting *A*. *thaliana* [22,23]. They reported that some *B*. *cinerea* sRNAs (siR3.1, siR3.2, siR5 and siR37) can mediate cross-kingdom repression of the plant immunity genes MPK1, MPK2, PRXIIF, WAK, ATG5, WRKY7, FEI2 and PMR6 by sequestration of the plant silencing protein AGO1. *B*. *cinerea* secretes extracellular vesicles loaded with fungal sRNAs which can be taken up by *A*. *thaliana* cells by endocytosis [22–24]. Cai et al. (2018) described that *A*. *thaliana* secondary siRNAs TAS1c-siR483 and TAS2-siR453 can also mediate a cross-kingdom gene repression [25]: these sRNAs are selectively loaded into plant extracellular vesicles and transported to *B*. *cinerea* cells, reducing fungal virulence by repressing vesicle-trafficking fungal genes [25].

Regarding the chronology of the *A*. *thaliana*–*B*. *cinerea* interaction, Windram et al. (2012) working with *A*. *thaliana* detached leaves treated with *B*. *cinerea* reported that from the germination of the conidiophores, the fungus had an initial continuous growth of hyphae until 20 hours post inoculation (hpi), and that the first visual symptoms (primary lesion) appear around this time [7]. They also reported that most of the changes in plant gene expression occurred by 24 hpi, when lesions remained small and localized. He et al. (2023) reported the presence of *B*. *cinerea* extracellular vesicles (those capable of loading fungal effector sRNAs) at the infection site in *A*. *thaliana* leaves at 10 hpi [24]. We have previously reported that, as early as 6 hpi with *B*. *cinerea*, *A*. *thaliana* leaves have already triggered defense responses including ROS accumulation [26] and changes in expression of the RLKs CERK1 and LYK5, of the RLP BAK1, of the RLCK BIK1 and of the MAPKs MPKKK3, MPKKK5, MKK5 and MPK6 [27].

To our knowledge, there are currently no transcriptome-wide studies on the regulation mediated by sRNAs during the early interaction of *B*. *cinerea* with a host plant. Moreover, most of these studies are limited to the analysis of specific plant miRNAs, often neglecting the other classes of plant sRNAs, besides *B*. *cinerea* sRNAs. The aim of this study was to identify small RNAs that could play a role in the early regulation of the interaction between *A*. *thaliana* and *B*. *cinerea*, analyzing the abundance of different classes of sRNAs isolated from leaf samples, comparing between control (mock treated) and *B*. *cinerea* treatments, using a high-throughput sequencing approach at 6 hpi. We performed a computational search for putative targets for the differentially expressed sRNAs between treatments. By integrating differentially expressed gene data from the same experimental conditions, we looked not only for contrasting expression profiles between treatments but also for contrasting expression profiles between sRNAs and their putative targets. The identification of such sRNA-mediated regulatory circuits could help develop sustainable strategies to mitigate fungal infection, which, ideally, could be applied not only in *A*. *thaliana* but also in other plants.

## Materials and methods

### Growth conditions and infection of *Arabidopsis thaliana* and *Botrytis cinerea*

We stratified *A*. *thaliana* seeds (ecotype C24) for four days at 4°C and later germinated them on a substrate consisting of a mixture of peat moss Sunshine #3 mix and vermiculite (3:1) under green-house conditions at 20–22°C with a 16-h light photoperiod until plants were four weeks old. We cultured *B*. *cinerea* (strain B05.10) on Potato Dextrose Agar (PDA, 39 g/L) for approximately 20 days at room temperature. To remove hyphae, we filtered the spores and harvested them in distilled water as previously described [28].

We treated four-week-old adult plants with *B*. *cinerea* spores or with a control treatment (mock). For *B*. *cinerea* treatment, we adjusted the spore suspension to a concentration of 5x10^4^ spores/mL in ¼ Potato Dextrose Broth medium (¼ PDB, 6 g/L), as in [27]. We incubated this concentration-adjusted suspension at room temperature for one hour before applying it to plants. For mock treatment, we used only ¼ PDB medium. We applied the treatments by spraying them over the entire surface of the plant leaves. To ensure spore germination, we kept the plants in complete darkness and high humidity conditions, covered them with plastic lids and placed them in a growth chamber at 22°C.

For each treatment, we collected approximately three fully expanded leaves per plant from 15 plants 6 hours after treatment application (6 hpi). We performed the experiment twice to have two independent biological replicates. Immediately, we froze the collected samples (~45 leaves per treatment per biological replicate) in liquid nitrogen, and we stored them at -80°C until further processing.

### RNA extraction, sequencing, and differential expression analysis

To isolate total RNA, we ground the frozen plant tissues into powder, and processed them according to the mirVana™ miRNA extraction kit (Invitrogen®) protocol. Subsequently, we sent the samples to Beijing Genomics Institute (BGI Americas) for the isolation of the small RNA (sRNA) fractions, library construction and sequencing for both total RNA and sRNAs.

We performed the data analysis corresponding to the total RNA based on [27]. Briefly, BGI performed library construction and sequencing by DNBSEQ™ sequencing technology. We aligned sequenced reads to the reference genome of *A*. *thaliana* (TAIR version 10) using Bowtie2 (v2.3.5) [29]. We calculated abundances of mapped reads using the RNA-seq by expectation maximization (RSEM) method (v1.3.3) [30].

For sRNA analysis, sRNA library construction and sequencing were performed by BGI using the DNBSEQ™ UMI Small RNA sequencing technology. BGI also computationally filtered the raw sequences by removing adapter, contaminating and low-quality sequences. Cleaned libraries consisted of more than 10 million reads each (S1 Table). Using these cleaned sequences, we performed an analysis to assess the quality of the data and checked the similarity between replicates in each treatment (S1 Fig). To assess whether we had recovered sequences corresponding to sRNAs in the clean reads, we analyzed the abundance distribution of the 18- to 25-nucleotide RNA sequences mapped to the *A*. *thaliana* genome. For these sequence alignments, we searched for perfect matches between our clean reads and the TAIR10.1 reference genome using Bowtie (v1.3.1). We observed that the 24- and 21-nucleotide sRNA species were the most abundant in both treatments (S2 Fig). This sRNA length distribution agrees with previous transcriptome-wide reports on *A*. *thaliana* and plant sRNAs [20,31–34].

We searched for known *A*. *thaliana* sRNA sequences from online resources and literature and produced a locally curated list that served as a reference for mapping the sRNA reads. Our reference list included the mature microRNA (miRNA) sequences deposited in miRBase (version 22.1) [35] and sRNAanno database [36], the sRNA sequences deposited in the Plant small RNA genes database [37] and the sequences of the sRNAs delivered by *A*. *thaliana* to *B*. *cinerea* via extracellular vesicles, reported by Cai et al. (2018) [25]. We also included the sequences of the sRNAs delivered by *B*. *cinerea* to *A*. *thaliana* reported by Weiberg et al. (2013) and Wang et al. (2017) [22,23]. In total, our reference list of known sRNA sequences contains 19,027 non-redundant sRNA sequences from *A*. *thaliana* and 74 non-redundant sRNA sequences from *B*. *cinerea*.

As sequencing depth has been previously reported to have an impact on the number of genes detected as differentially expressed [38], our libraries sequencing depths were examined for differential expression analysis. We randomly subsampled reads from our cleaned libraries and evaluated the number of new sRNAs detected as subsampled reads were added. We reached a saturation plateau for sRNAs annotated as miRNAs and secondary siRNAs (S3 Fig). Similar sequencing depths have been reported by previous studies on responsive plant miRNAs to *B*. *cinerea* [20,33].

Using home-made scripts, we computationally mapped the cleaned sRNA reads for each of the four sequencing libraries (direct comparison between two biological replicates for 6 hpi mock-treated leaves and two biological replicates for 6 hpi *B*. *cinerea*-treated leaves) to our reference list of known sRNAs, allowing only for perfect matches and calculated abundances of the known sRNAs (a matrix of the number of raw counts for each library). For the differential gene expression analysis, we used these data counts as input for the DESeq2 software package [39]. DESeq2 takes such matrices of raw counts as input (no particular treatment for data counts from any source, including sRNA sequences), internally adjusts for library sizes, estimates dispersions and fold changes incorporating data-driven prior distributions and uses negative binomial generalized linear models to test for differential expression [39]. We used the “DESeq” function of DESeq2 to perform the estimation steps, the Wald test for hypothesis testing and the Benjamini–Hochberg False Discovery Rate (FDR) procedure for multiple comparisons adjustment for the identification of differentially expressed sRNAs, as well as mRNAs, between the *B*. *cinerea*- and mock-treated samples. We used the DESeq2 “results” function to obtain result tables considering an adjusted p-value ≤ 0.05 as cutoff. We only considered those RNAs that had at least 10 read counts in both biological replicates in at least one of the two experimental conditions. We did not detect any *B*. *cinerea* sRNA accumulation.

To validate our transcriptome-wide sRNA expression results using independent leaf samples, we performed stem-loop RT-qPCR experiments on selected sRNAs to confirm their expression patterns. We selected them because they or their targets have previously been implicated in plant immunity [34,40]. Total RNA samples obtained from mock and *B*. *cinerea* treated leaves (6 hpi) were used to determine miRNA accumulation by RT‐qPCR. For each treatment, a total of fifteen leaves were used (five leaves from each of three plants). Stem-loop RT-qPCR was performed as previously described [41]. Briefly, pulse stem-loop reverse transcription was performed for each selected sRNA and U6 snRNA (internal housekeeping control) using oligos listed in S2 Table. qPCR was performed using the 2^-ΔΔCt^ method to calculate relative expressions with mock treatment as reference. Two-tailed independent samples t-tests were performed and p-values <0.05 were considered significant.

For all the expressed sRNAs in our experimental conditions, we manually curated their sequence annotations according to the class of sRNA to which they belonged. To curate the annotations, we aligned each sequence of expressed sRNA to the *A*. *thaliana* reference genome (TAIR version 10) using the “TAIR10 Intergenic (DNA)” and “TAIR10 Genes (+introns, +UTRs) (DNA)” databases as search sets in the TAIR-BLAST 2.9.0+ web server (https://www.arabidopsis.org/Blast/, with default parameters), to later perform an exploration in genome browser for the location and genomic context of hits with perfect matches. The curated annotations consisted of the following tags: “miRNA”, “putative hpRNA”, “secondary siRNA” (which included ta-siRNAs and pha-siRNAs), “hc-siRNA” (heterochromatic siRNAs), tsRNA (tRNA-derived sRNA) and "other" (for sequences that did not belong to any of the other groups due to insufficient information).

### Computational inference of small RNA targets and integration of RNA-seq and small RNA-seq data

We performed computational inference of putative targets for the differentially expressed sRNAs using the psRNATarget web server (https://www.zhaolab.org/psRNATarget/) [42,43]. We used the “*Arabidopsis thaliana*, transcript, removed miRNA gene, TAIR, version 10, released on 2010_12_14” cDNA sequence library available on the server to search for putative targets. We performed target prediction with default parameters using the second version of the psRNATarget scoring scheme and a value of Expectation ≤ 3 as cutoff to filter the putative targets [43]. Using home-made scripts, we selected only the putative target sequences that appeared in the list of differentially expressed mRNAs and searched for an inverse correlation in the expression profiles between the differentially expressed sRNAs and their differentially expressed putative targets.

### GO term enrichment analysis for inferred targets

We performed a GO term enrichment analysis for the differentially expressed putative targets inferred for miRNAs, secondary siRNAs and tsRNAs; the sRNAs that most commonly exert their regulation at the post-transcriptional level. We carried out this analysis following a protocol designed for the visualization of enriched GO terms (https://bio-protocol.org/bio101/e3429). Briefly, we searched for enriched GO terms in the “Biological process” category, using the “Go Term Enrichment for Plants” tool from the online *A*. *thaliana* database TAIR (The Arabidopsis Information Resource). We used the *A*. *thaliana* gene list available on the server as reference for the algorithm. The tool utilizes PANTHER Classification System (Protein ANalysis THrough Evolutionary Relationships) (version 16.0) to run the analysis. We only considered those terms with a fold enrichment > 1 and a Benjamini-Hochberg false discovery rate value (FDR) < 0.05 calculated over Fisher exact test values. For these enriched GO terms, we performed an analysis to avoid redundancy within the terms using REVIGO (http://revigo.irb.hr/) using the *A*. *thaliana* GO term association frequencies reference database. We only considered those terms with a relevance similarity score (SimRel) < 0.05.

## Results

### *Arabidopsis thaliana* sRNAs responded to the fungal pathogen *Botrytis cinerea* at an early stage of interaction

**Fig 1:**
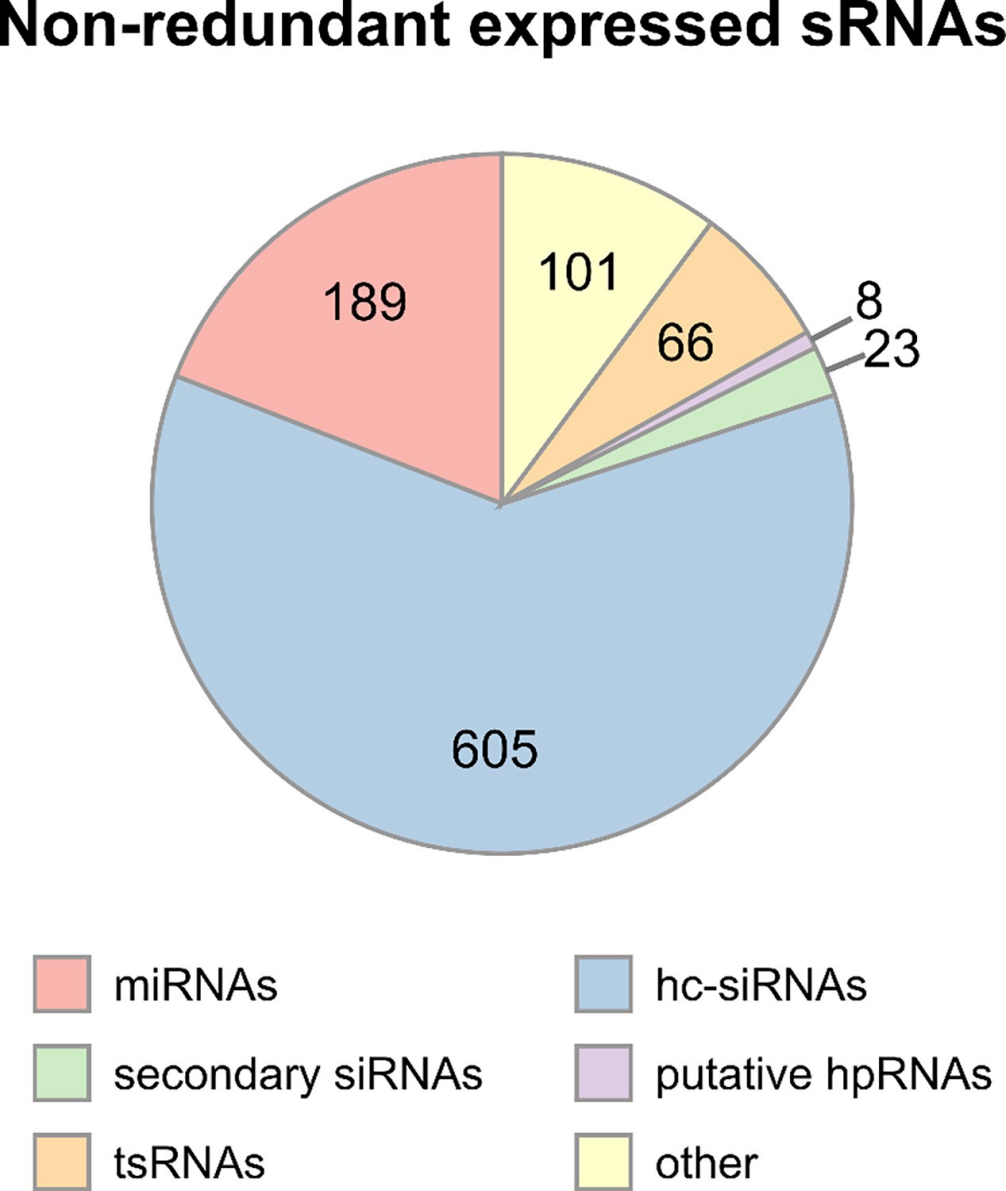
Abundances of the different classes of non-redundant plant sRNAs. Using DESeq2 for expression analysis, we found a total of 992 non-redundant expressed sRNAs that were present in our four libraries. The classes identified were heterochromatic siRNAs (hc-siRNAs), miRNAs, secondary siRNAs, putative hpRNAs and tRNA-derived sRNAs (tsRNAs) among the expressed sRNAs and other short sequences that did not correspond to any of the other classes. Numbers represent counts of non-redundant plant sRNAs.

To identify responsive sRNAs with potential regulatory roles in the early interaction between *A*. *thaliana* and *B*. *cinerea* using next generation sequencing, we analyze the expression profiles of the sRNAs from four-week-old plants to which we had applied mock or *B*. *cinerea* treatments. In our analysis, we quantified and annotated all expressed sRNAs and obtained a total of 992 non-redundant sRNA sequences expressed in both conditions (S3 Table) that belonged to different plant sRNAs classes: 605 heterochromatic siRNAs (hc-siRNAs) (~61% of all expressed sRNAs), 189 miRNAs (~19%), 23 secondary siRNAs (~2.3%), 66 tRNA-derived sRNAs (tsRNAs) (~6.6%), 8 putative hpRNAs (~0.8%) and other 101 short sequences that did not correspond to any of the other sRNA classes (~10.2%) (Fig 1). We identified 325 differentially expressed sRNAs (185 upregulated and 140 downregulated) from *B*. *cinerea* treatment relative to mock treated plants (S4 Table).

**Fig 2:**
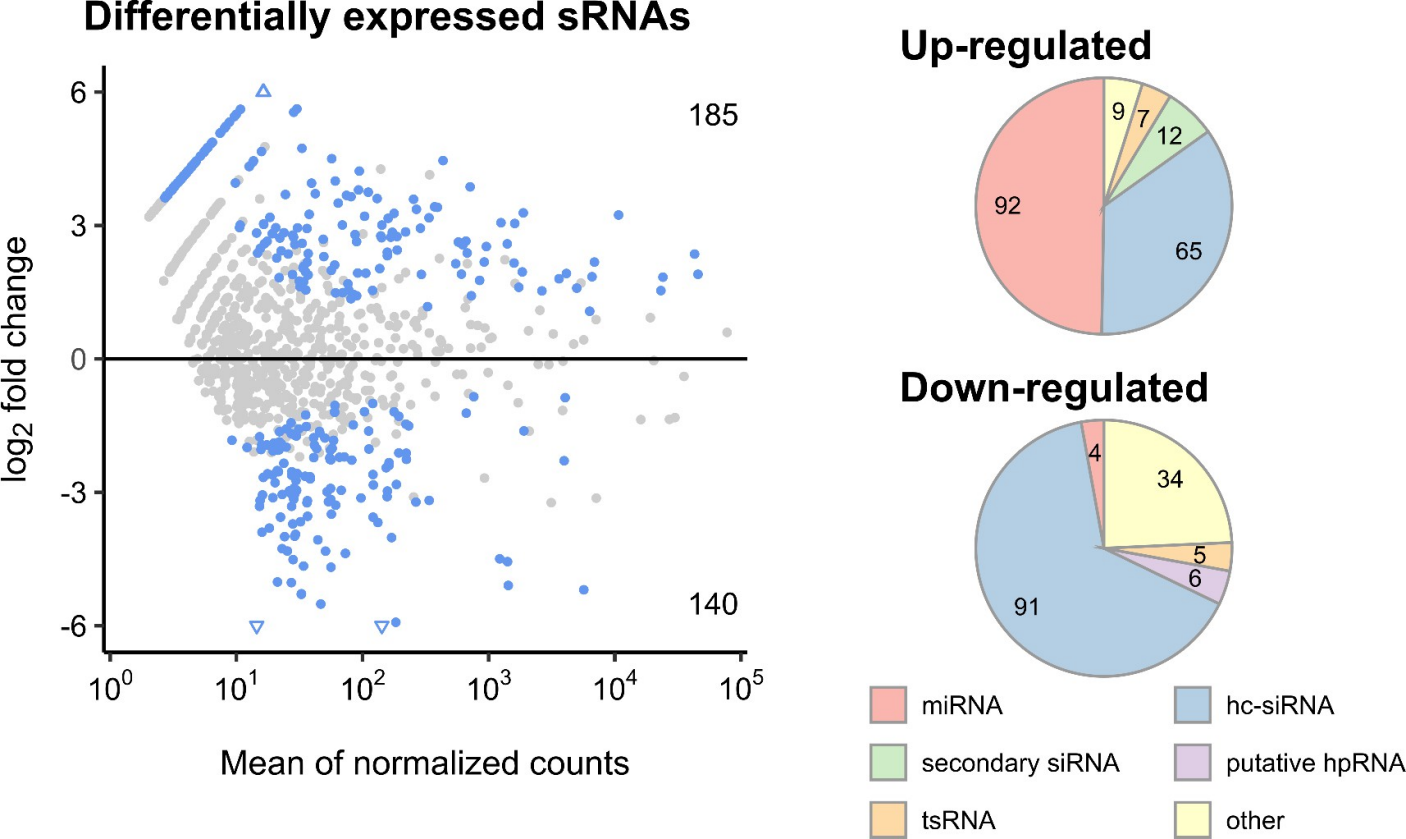
Differentially expressed sRNAs upon *B*. *cinerea* infection. The left graph shows differentially expressed sRNAs between treatments (*B*. *cinerea* compared to mock). Blue and grey dots are sRNAs with and without statistically significant differences in expression, respectively, (adjusted p-value ≤ 0.05, as obtained from differential expression analysis using DESeq2). The numbers at the corners indicate upregulated (above) or downregulated (bottom) sRNAs. The right graphs show the sRNA classes abundances of upregulated (above) and downregulated (bottom) sRNAs. Numbers in pie charts represent counts of non-redundant plant sRNAs.

Interestingly, almost half the upregulated sRNAs were miRNAs, around 35% belonged to hc-siRNAs, 6% were secondary siRNAs, 3% were tsRNAs and the remaining 4% corresponded to other sequences. In contrast, 65% of the downregulated sRNAs were hc-siRNAs, 24% corresponded to other sequences, 3% were tsRNAs, only 2% corresponded to miRNAs and the remaining 6% were putative hpRNAs (Fig 2). To validate our transcriptome-wide sRNA expression results using independent leaf samples, we performed stem-loop RT-qPCR experiments on selected sRNAs to confirm their expression patterns (S4 Table and S4 Fig). We analyzed the accumulation of miR403-3p and miR167b/a-5p/c since they or their targets have previously been implicated in plant immunity [34,40]. For all the selected sRNAs we confirmed that expression patterns of selected sRNAs matched those found in our transcriptome-wide analysis (S4 Table and S4 Fig).

### Evolutionary conserved miRNAs were the most accumulated *A*. *thaliana* sRNAs from the different classes of differentially expressed sRNAs in response to *B*. *cinerea* at an early stage of interaction

**Fig 3:**
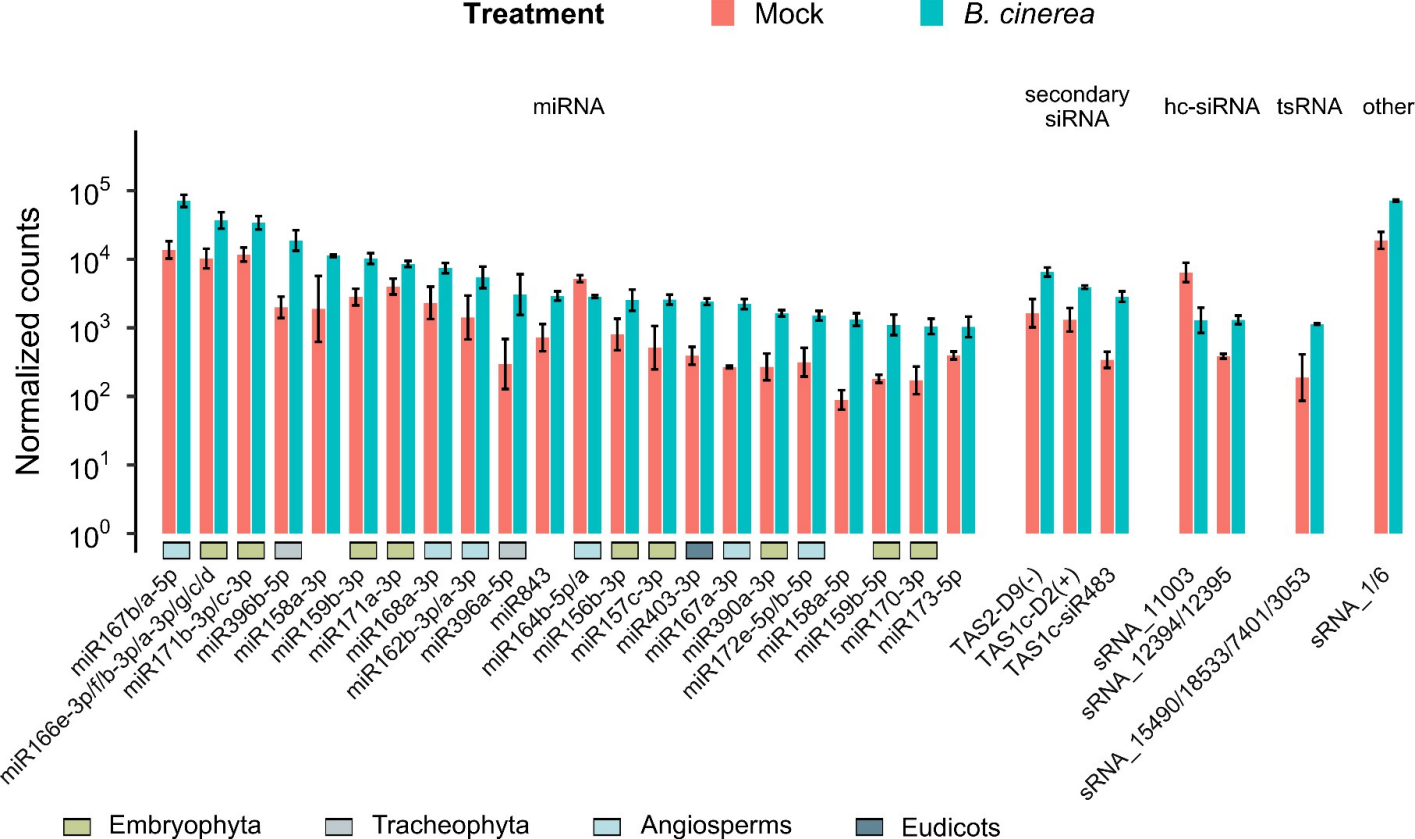
Evolutionarily conserved miRNAs and some known *B*. *cinerea-*responsive *TAS* loci-derived secondary siRNAs represented the majority of the differentially expressed *A*. *thaliana* sRNAs among the most accumulated in response to *B*. *cinerea* at 6 hpi. Some of these miRNAs are conserved in the Embryophyta (green), Trachaeophyta (gray), Angiosperms (blue) and Eudicots (dark green) plant lineages. Bar plot shows all differentially expressed sRNAs (adjusted p-value ≤ 0.05, as obtained from differential expression analysis using DESeq2) which had at least an average of 1000 normalized counts in the *B*. *cinerea* condition. Normalized counts were obtained using the DESeq2 algorithm. Bar plot indicates the mean of the normalized expression values of two biological replicates, the error bars represent the standard deviation.

We checked which differentially expressed sRNAs were the most accumulated in plants treated with *B*. *cinerea*. Among them, the miRNAs that showed upregulated expression were miR167b/a-5p and miR167a-3p, miR166e-3p/f/b-3p/g/c/d, miR170-3p, miR171b-3p/c-3p, miR171a-3p, miR396b-5p, miR396a-5p, miR159b-3p and miR159b-5p, miR156b-3p, miR403-3p, miR172e-5p/b-5p, miR158a-3p and miR158a-5p, miR168a-3p, miR162b-3p/a-3p, miR843, miR157c-3p, miR390a-3p and miR173-5p. Although highly expressed, miR164b-5p/a showed downregulation during *B*. *cinerea* infection. Almost all *B*. *cinerea*-responsive and highly abundant miRNAs were conserved in Embryophyta (land plants), Tracheophyta (vascular plants), Angiosperms (flowering plants) and Eudicots (flowering plants, commonly known as dicots), except miR173, miR158 and miR843, which are non-conserved miRNAs (Fig 3). Furthermore, an RNA sequence annotated as sRNA_1/6, a tsRNA annotated as sRNA_15490/18533/7401/3053, two hc-siRNAs sRNA_11003 and sRNA_12394/12395, and three secondary siRNAs: TAS2-D9(-), TAS1c-D2(+), TAS1c-siR483 were highly abundant in response to *B*. *cinerea* infection (Fig 3). Given the abundance and differential expression of these sRNAs, these results indicate that the early response of A. *thaliana* to *B*. *cinerea* infection is characterized by the upregulation and high expression of evolutionarily conserved miRNAs.

### Known pathogen-responsive miRNAs and secondary siRNAs accumulated in response to *B*. *cinerea*

**Fig 4:**
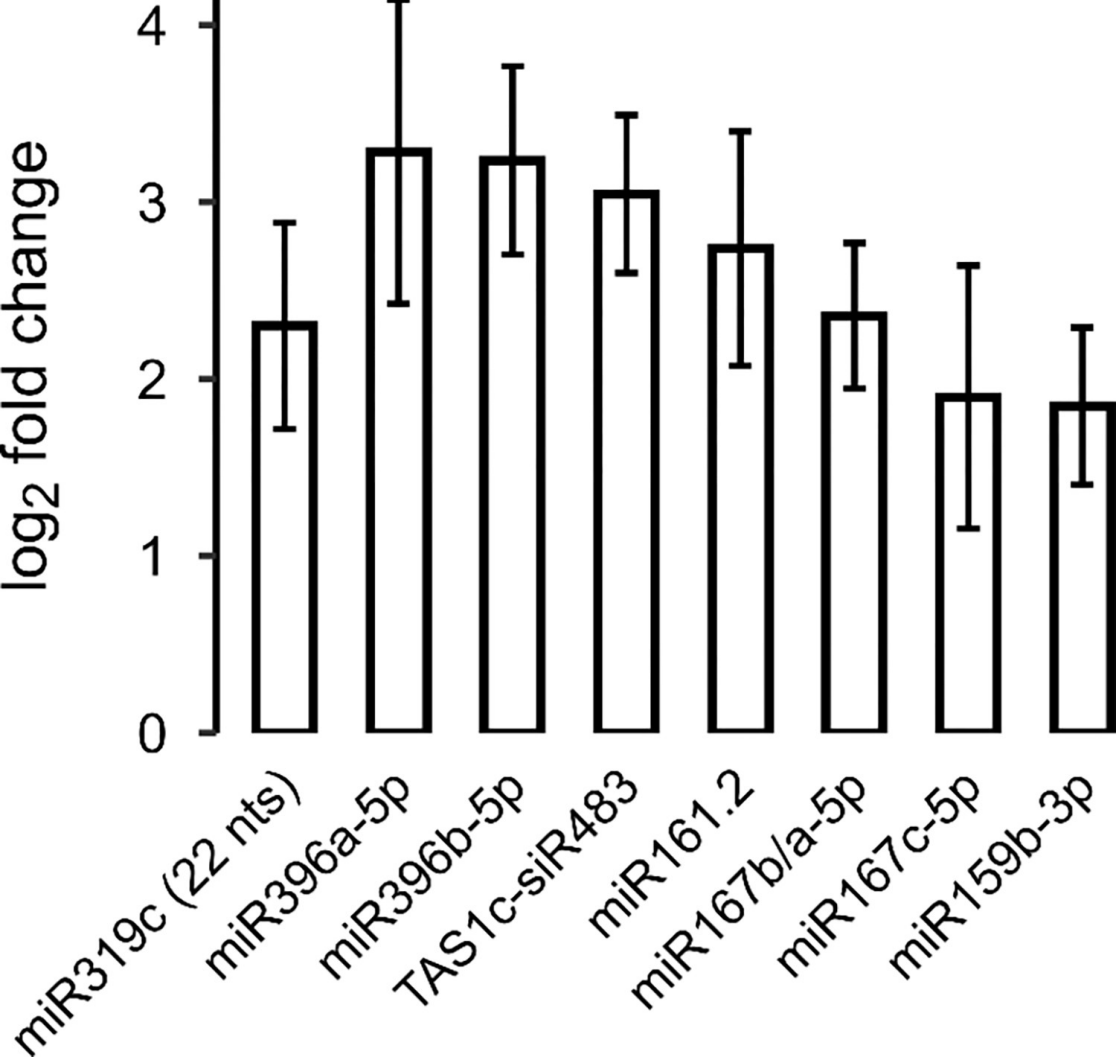
Differentially expressed known pathogen responsive sRNAs at 6 hpi with *B*. *cinerea* treatment. Bars indicate the fold change of the expression levels of the differential expressed sRNAs (adjusted p-value ≤ 0.05, as obtained from differential expression analysis using DESeq2) miR319c (22 nts), miR396a-5p, miR396b-5p, TAS1c-siR483, miR161.2, miR167b/a, miR167c-5p and miR159b-3p in *B*. *cinerea* treatment compared to mock, the error bars represent standard error.

Recent studies have previously reported plant small RNAs that could be key in response to pathogenic bacteria, fungi, and oomycetes such as *Pseudomonas syringae*, *Plectosphaerella cucumerina*, *Botrytis cinerea* and *Phytophthora capsici* [25,31,34,44]. In response to *B*. *cinerea*, we found the early upregulation of some of these known pathogen-responsive sRNAs: miR319c (22 nts), miR396a-5p, miR396b-5p, miR161.2, miR167b/a, miR167c-5p and miR159b-3p, and the secondary siRNA TAS1c-siR483 (Fig 4). miR167, miR159 and miR319c regulate transcripts of genes involved in the biosynthesis and signaling pathways of the plant hormones auxin, abscisic acid and jasmonic acid in *A*. *thaliana* [34]. Therefore, we checked whether the expression profiles of miR167, miR159, miR319c target genes exhibited downregulation in our data following mock and *B*. *cinerea* treatments. The Auxin Response Factor 8 (ARF8) gene, targeted by miR167, was downregulated during *B*. *cinerea* infection (S5 Fig). Regarding miR159 target genes, MYB65 did not show any changes in its expression profile, and we could not detect MYB101 expression. However, one isoform of MYB33 (AT5G06100.3) was downregulated in *B*. *cinerea* treatment at 6 hpi (S5 Fig). miR319 targets transcripts of the TCP gene family, which encodes transcription factors primarily related to development, and control of the biosynthesis of the plant hormone jasmonic acid [45,46]. TCP2 (AT4G18390.1 and AT4G18390.2) and TCP10 (AT2G31070.1), both target genes of miR319c, were also downregulated upon *B*. *cinerea* infection (S5 Fig). This suggests that these miRNAs could be involved in the regulation of immunity by indirectly fine-tuning the plant hormones auxin, abscisic acid and jasmonic acid in *A*. *thaliana* during *B*. *cinerea* infection.

**Fig 5:**
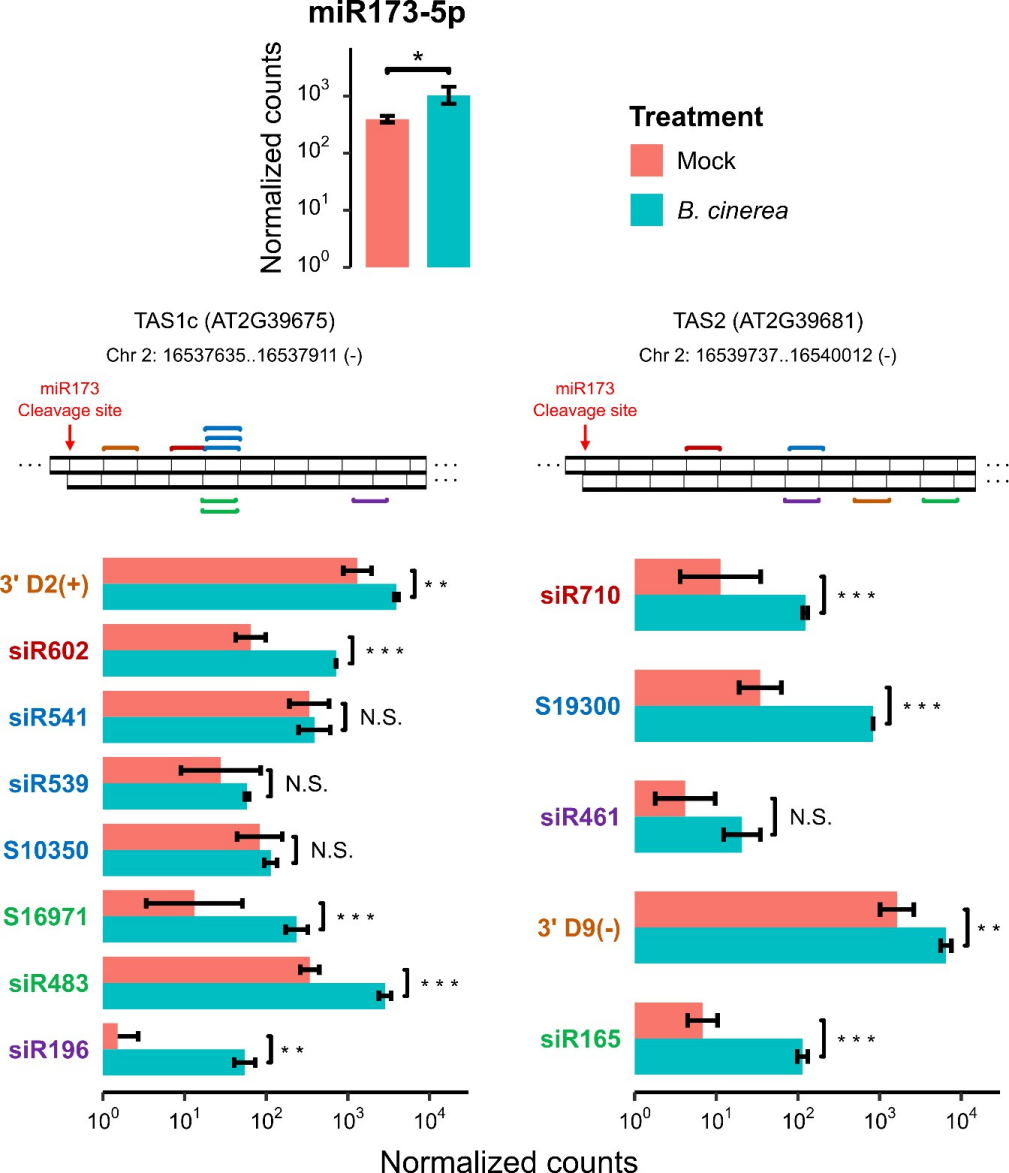
miR173-5p as well as some known *A*. *thaliana* extracellular vesicle-loaded TAS-derived secondary siRNAs are upregulated during early *B*. *cinerea* infection. TAS derived-secondary siRNAs that are loaded in *A*. *thaliana* extracellular vesicles are indicated with different colors in the respective dsRNA diagrams. The plot shows the expression levels of miR173-5p and TAS1c and TAS2-derived secondary siRNAs between mock and *B*. *cinerea* treatments. Normalized counts of expression values were obtained using the DESeq2 algorithm. Error bars represent standard deviation of two biological replicates. Differential expression between mock and *B*. *cinerea* treatment is indicated by asterisks (adjusted p-value < 0.05 (*), < 0.01 (**) and < 0.001 (***), or no significant (N.S.), as obtained from differential expression analysis using DESeq2).

On the other hand, Cai et al., (2018) described that TAS1c-siR483 and TAS2-siR453 (secondary siRNAs derived from TAS1c and TAS2 *A*. *thaliana* loci), were selectively loaded into plant extracellular vesicles, transported to the *B*. *cinerea* cells, and reduced fungal virulence by mediating cross-kingdom repression of vesicle-trafficking genes [25]. Interestingly, we found that miR173, which triggered the production of secondary siRNAs from TAS1a, TAS1b, TAS1c and TAS2 [47,48], was slightly upregulated upon *B*. *cinerea* treatment (6 hpi) (Fig 5). We also found the upregulation of secondary siRNAs derived from TAS1c and TAS2 transcripts during *B*. *cinerea* treatment (S6 and S7 Figs and Fig 5). We detected the upregulation of the TAS1c-derived secondary siRNAs TAS1c-3’ D2 (+) (highly abundant), siR602, S16971, siR483 (highly abundant) and siR196, and although we did not detect expression of TAS2-siR453 in any of the treatments we did detect the upregulation of the TAS2-derived secondary siRNAs siR710, S19300, TAS2-3’ D9 (-) (highly abundant) and siR165 with *B*. *cinerea* treatment compared to mock (Fig 5). Taken together, these results indicate that sRNAs transported into *B*. *cinerea* cells via extracellular vesicles produced by *A*. *thaliana*, are already induced in the plant at this time point of the interaction (6 hpi).

### Putative mRNA targets for the differentially expressed sRNAs

To evaluate the processes in which the differentially expressed sRNAs are involved, we performed a computational analysis using psRNATarget to search for putative mRNA targets for these sRNAs. Using the data from our previous publication where we worked on the transcriptome characterization of *A*. *thaliana* in the same conditions of mock and *B*. *cinerea* treatment at 6 hpi [27], we searched for the differentially expressed mRNAs and found 6,247 and 5,997 upregulated and downregulated mRNAs, respectively (S5 and S6 Tables and S8 Fig). We selected only those sRNAs and their putative mRNA targets that showed an inverse correlation in their expression profiles. We found 499 cases exhibiting this type of correlation. Integrated results of differentially expressed sRNAs and anticorrelated differentially expressed mRNA inferred targets are available in S7 Table.

**Fig 6:**
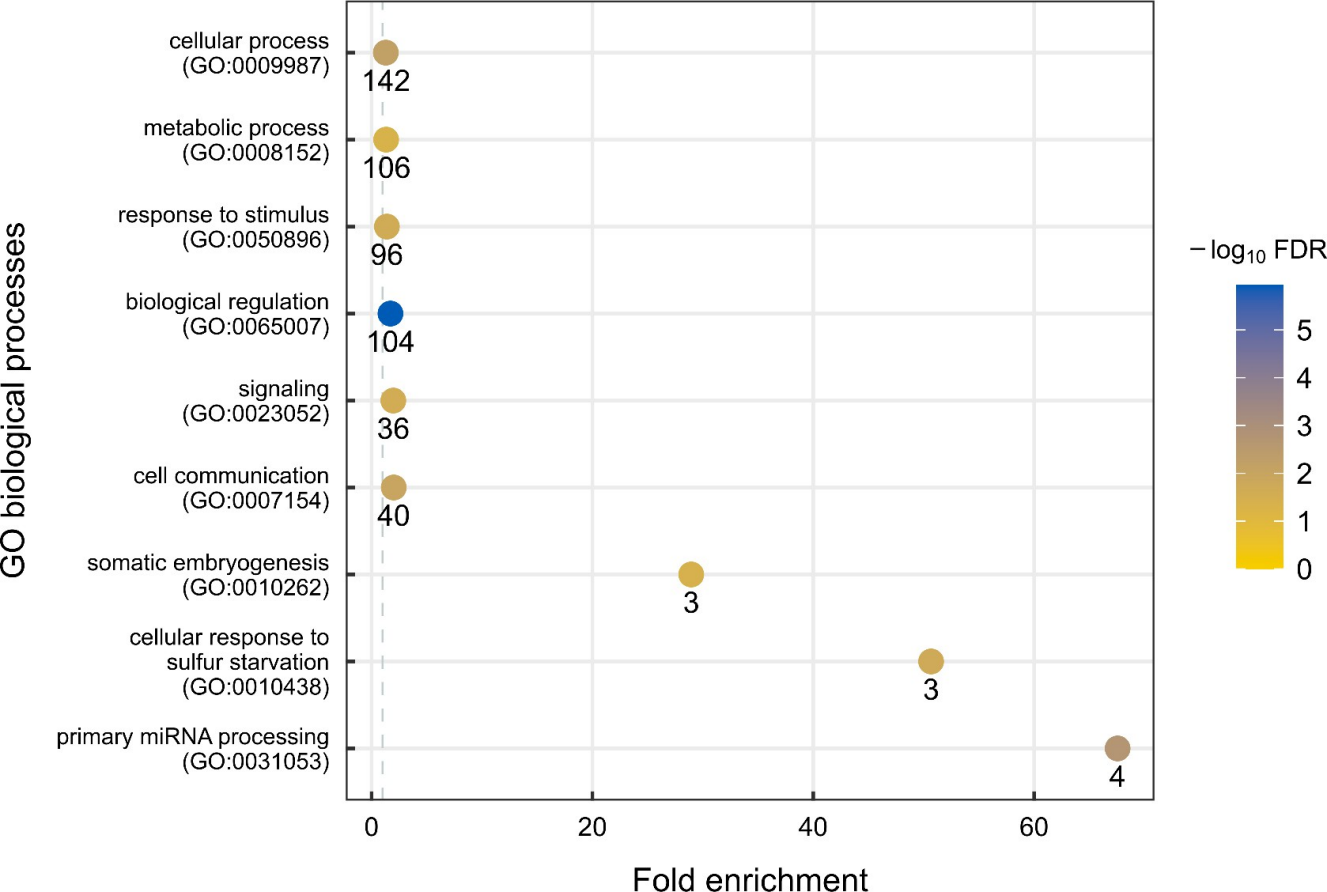
Biological processes in which the putative mRNA targets for sRNAs (miRNAs, secondary siRNAs, and tsRNAs) are involved. The putative mRNA targets used in the GO term enrichment analysis have contrasting patterns of expression with their corresponding regulatory sRNAs. The plot shows the enriched GO terms for biological process (FDR < 0.05), the color of the circles indicates the FDR value of each enriched term and the number of genes associated with each term is indicated under each circle.

To identify the biological processes in which *A*. *thaliana* differentially expressed sRNAs and their putative mRNA targets during *B*. *cinerea* infection, we performed a Gene Ontology (GO) term enrichment analysis. The latter included target transcripts of miRNAs, secondary siRNAs, and tsRNAs with contrasting expression patterns. In general, we found the enrichment of GO terms related to different processes, such as biological regulation, cellular and metabolic processes, response to stimulus, signaling, cell communication, somatic embryogenesis, cellular response to sulfur starvation and primary miRNA processing (Fig 6). The enriched GO terms and the respective associated genes are presented in S8 Table.

### miRNA-mediated regulation of DCL1 and AGO2 as part of the early response to *B*. *cinerea*

**Fig 7:**
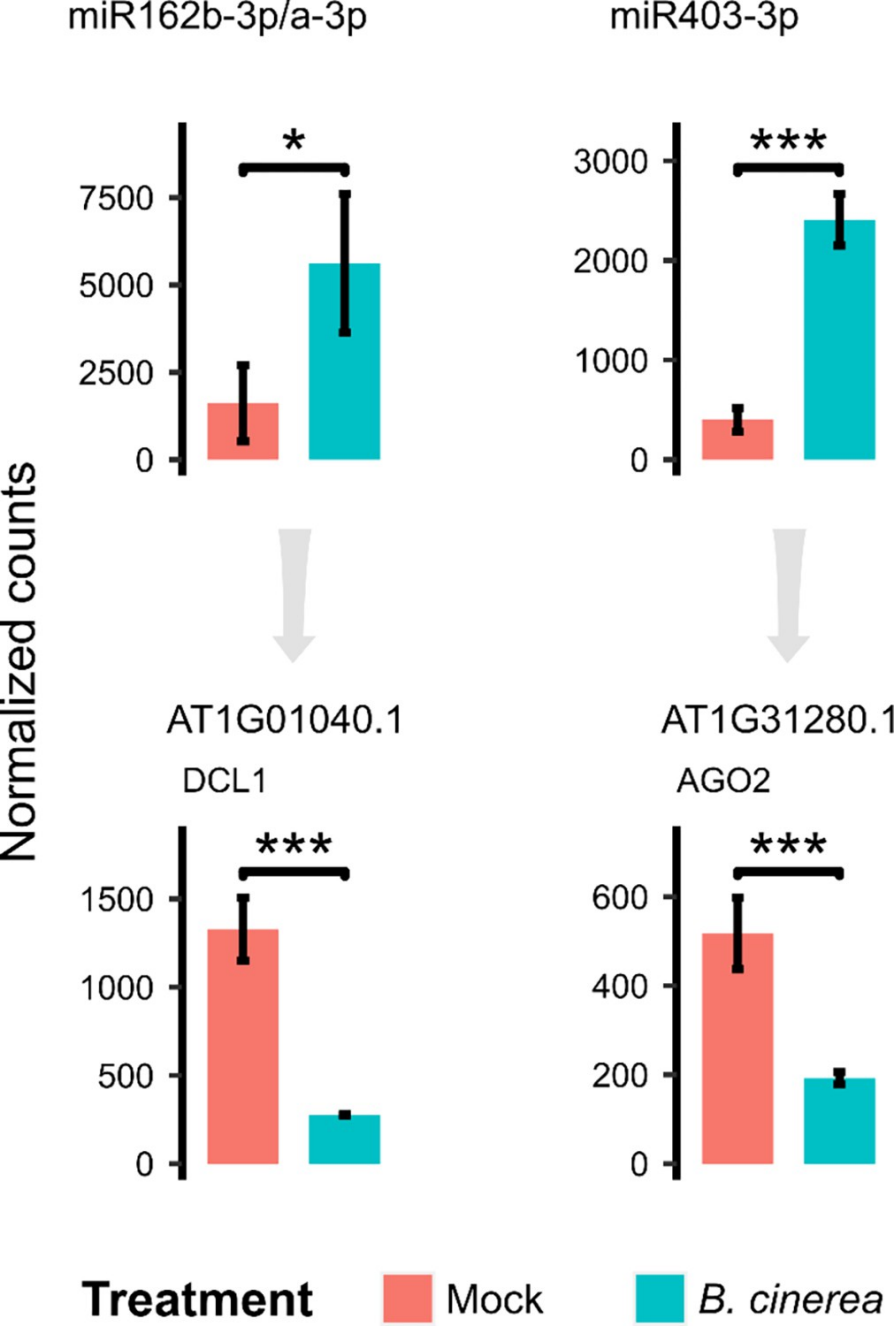
small RNA-mediated gene silencing genes and their regulatory sRNAs are regulated during early *B*. *cinerea* infection. Expression profiles of miR162b-3p/a-3p and *DCL1* (left) and expression profiles of miR403-3p and *AGO2* (right) between mock and *B*. *cinerea* treatments. Normalized counts were obtained using DESeq2. Error bars represent standard deviation of two biological replicates. Differential expression between mock and *B*. *cinerea* treatment is indicated by asterisks (adjusted p-value < 0.05 (*) and < 0.001 (***), as obtained from differential expression analysis using DESeq2).

Interestingly, the GO term that was enriched the most was “primary miRNA processing (GO:0031053)” (Fig 6). One of the four target genes associated with this term was *DCL1* (AT1G01040), a gene whose protein product is an enzyme involved in the biogenesis of miRNAs, specifically in the processing of primary transcripts and the production of mature miRNAs [49].The twenty-exon full-length *DCL1* mRNA can be post-transcriptionally regulated by miR162, with the corresponding cleavage site formed by the junction of exons 12 and 13 [50]. In our analysis, miR162b-3p/a-3p was upregulated and negatively correlated with the downregulation of *DCL1* transcripts in the *B*. *cinerea* treatment (Fig 7). Furthermore, the enriched GO term “response to stimulus (GO:0050896)” was associated with another sRNA-mediated gene silencing related gene: *AGO2* (AT1G31280). It has been described that *AGO2* is an RNAi effector gene involved in plant antiviral defense, and its transcripts can be regulated by miR403 [40,47]. In our analysis, miR403-3p was upregulated and *AGO2* was downregulated by *B*. *cinerea* infection (Fig 7). Taken together, these results suggest that post-transcriptional regulation of *DCL1* and *AGO2* transcripts, and thus sRNA-mediated silencing pathways, could be important for *A*. *thaliana* to mediate the early response to *B*. *cinerea*.

### miR161.2, miR400, TAS2-siR165 and a subset of PPR genes respond to *B*. *cinerea* at an early stage of the infection

**Fig 8:**
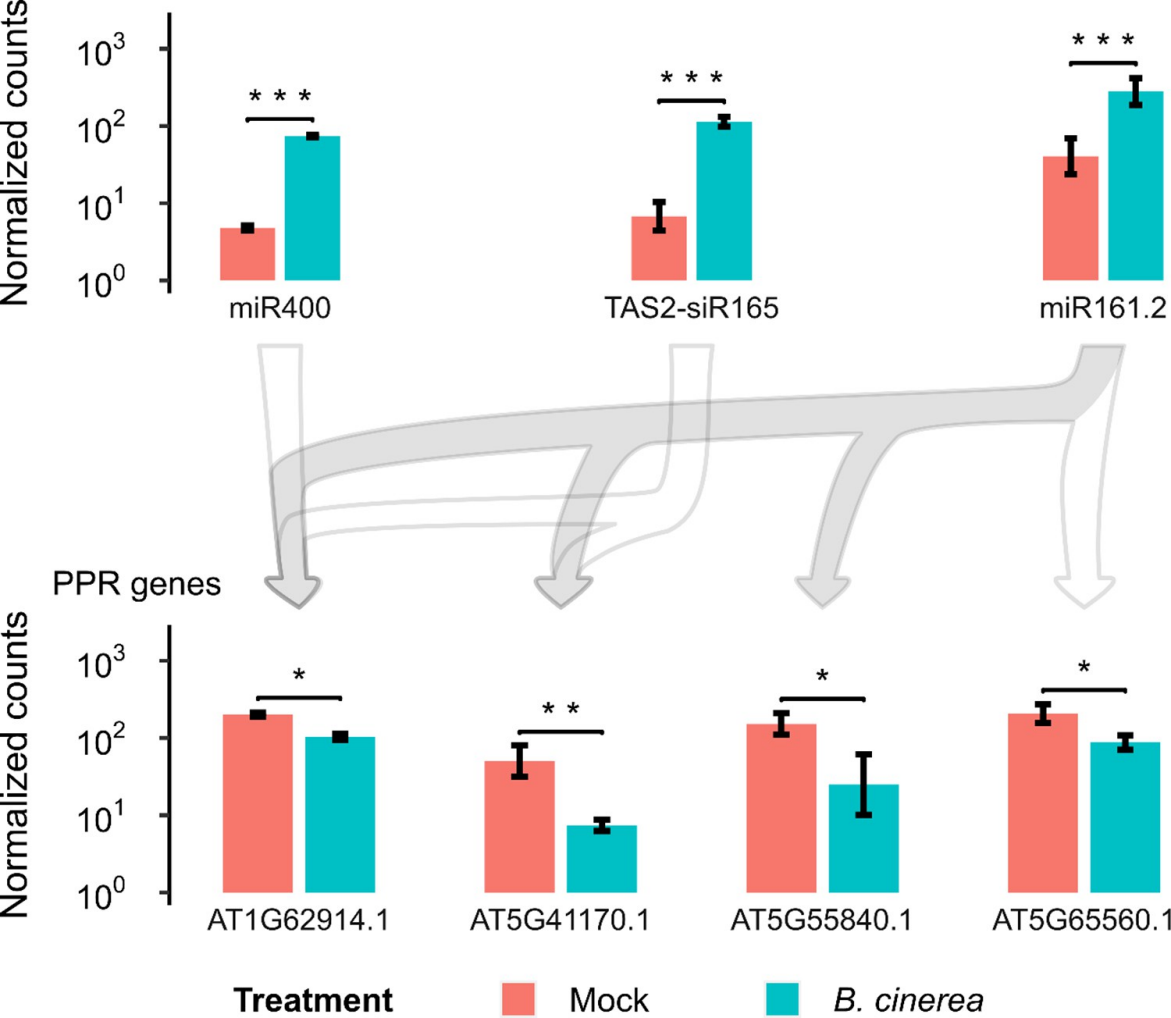
Regulation of the expression of miR161.2, miR400, TAS2-siR165 and their respective PPR gene targets during early *B*. *cinerea* infection. Normalized counts of expression values were obtained using DESeq2. Error bars represent standard deviation of two biological replicates. Differential expression between mock and *B*. *cinerea* treatment is indicated by asterisks (adjusted p-value < 0.05 (*), < 0.01 (**) and < 0.001 (***), or no significant (N.S.), as obtained from differential expression analysis using DESeq2). Filled and empty arrows connect sRNAs with confirmed and putative PPR target genes, respectively.

One of the genes associated with the GO term “biological regulation (GO:0065007)” was AT5G55840, which belongs to the Pentatricopeptide repeat (PPR) gene superfamily. Some PPR proteins perform their functions mainly in mitochondria or chloroplasts, binding one or several organellar transcripts, and influencing their expression by altering RNA sequence, turnover, processing, or translation [51]. PPR genes are post-transcriptionally regulated by sRNAs and could be involved in the regulation of the *A*. *thaliana* immune response. Park et al. (2014) reported that miR400 regulates AT1G62720 PPR gene and knockdown mutants of this PPR gene are more susceptible to *B*. *cinerea* [52]. The sRNAs miR161.1, miR161.2, miR400 and some TAS1a/b/c- and TAS2-derived secondary siRNAs (which in turn are produced after miR173-mediated cleavage of their TAS precursor transcripts) can regulate several *A*. *thaliana* PPR genes [52–54]. AT5G55840 together with AT1G62914, AT5G41170 and AT5G65560 were among the differentially expressed PPR genes, which were predicted to be targets of miR161.2 (S7 Table). The cleavage of AT1G62914 and AT5G41170 by miR161.2 has been previously reported [53–55], whereas the cleavage of AT5G65560 has not yet been reported. In our laboratory we have confirmed the cleavage-mediated regulation by miR161.2 of AT5G55840 (Ana Karen Avila-Sandoval, personal communication, June 2023). In addition to miR161.2 cleavage, we also found that AT1G62720 and AT1G62914 PPR genes were regulated by miR400 and TAS2-siR165, with the latter also regulating AT5G41170 (S7 Table). In our *B*. *cinerea*-treated plants, miR161.2, miR400 and TAS2-siR165 were up-regulated (Figs 5 and 8) and, although AT1G62720 was not differentially expressed between the treatments, we found that AT1G62914, AT5G41170, AT5G55840 and AT5G65560 transcripts were downregulated compared to mock-treated plants (Fig 8). These results suggest that miR161.2, miR400 and miR173/TAS2-siR165 mediate the regulation of a subset of *PPR* genes during early *B*. *cinerea* infection in *A*. *thaliana*.

## Discussion

Weiberg et al. (2013) analyzed libraries of sRNAs extracted from samples of *A*. *thaliana* leaves treated with *B*. *cinerea* at 24, 48 and 72 hpi and reported that *B*. *cinerea* sRNAs participate as effectors in the interaction with *A*. *thaliana*, repressing plant defense genes (cross-kingdom regulation) [22]. He et al. (2023) reported that *B*. *cinerea* extracellular vesicles (the type of vesicles that transport the effector sRNAs of this fungus) were already present at 10 hpi at the site of infection in *A*. *thaliana* leaves, according to microscopy analysis [24]. In our work, we did not detect the accumulation of known *B*. *cinerea* sRNAs involved in cross-kingdom regulation at an early stage of infection (6 hpi). This is probably because 6 hpi is a very early time point in the interaction in which *B*. *cinerea* has not produced those sRNAs yet or not in sufficient quantity to be detected. Nevertheless, we found that different classes of plant sRNAs (hc-siRNAs, miRNAs, secondary siRNAs, tsRNAs and other putative hpRNAs) were responsive to *B*. *cinerea* at this early time point of the interaction. Windram et al. (2012) reported that *A*. *thaliana* leaves treated with *B*. *cinerea* undergo dynamic changes in expression of transcription factors and of genes related to biosynthesis and signaling of plant hormones, highlighting the value of having fine temporal analysis [7]. Further research is needed to explore the dynamic expression changes of miRNAs and secondary siRNAs throughout the infection process, with a focus on understanding their potential regulatory roles in mRNA accumulation dynamics. Additionally, as the infection progresses and fungal biomass increases, it is expected that sRNA sequences from *B*. *cinerea* will be increasingly detectable. Given that this study focuses only on previously reported sRNA sequences from *B*. *cinerea* and *A*. *thaliana*, more investigation is also crucial to assess for other *B*. *cinerea* undiscovered sRNAs that can also mediate cross-kingdom regulations, as well as for determining the timing of their production.

### Early contribution of hc-siRNAs in the *A*. *thaliana* response to *B*. *cinerea*

Mostly generated from transposons, repetitive and heterochromatic regions in the plant genome, hc-siRNAs commonly silence transposons at a transcriptional level by regulating DNA methylation and histone modification, thus contributing to genome stability [56,57]. Demethylation pathways actively antagonizes the DNA methylation machinery to prevent the spread of DNA methylation from target loci [58]. A link between DNA demethylation and activation of plant immunity has been reported, which may be caused by a greater ease of expression of defense related genes possibly resulting from two common responses to phytopathogens: DNA hypomethylation and subsequent chromatin relaxation [12,58]. From an evolutionary perspective, these epigenetic changes may facilitate the mobilization of transposons to pathogen-responsive genes, thereby contributing to gene diversification and adaptation of the plant immune system [58]. In agreement with previous findings on tomato leaves where the downregulation of the 24-nucleotide population of sRNAs in response to *B*. *cinerea* was reported [20], we found that hc-siRNAs represented an important proportion from the non-redundant differentially expressed sRNAs at 6 hpi, accounting for 65% of the downregulated sRNAs with *B*. *cinerea* treatment. Further research is needed to explore the expression dynamics of hc-siRNAs throughout the infection process, with a focus on their potential regulatory roles in the regulation of the DNA methylation dynamics. Thus, an early downregulation of the hc-siRNAs in *A*. *thaliana* could be an important mechanism during the interaction with *B*. *cinerea*, allowing the plant to enhance the expression of defense related genes. Given that an important proportion of the differentially expressed sRNAs correspond to hc-siRNAs, further investigation is needed to understand the role of these sRNAs in the regulation of the *A*. *thaliana* and *B*. *cinerea* interaction.

### Early expression regulation of plant hormone regulatory miRNAs during *B*. *cinerea* infection

Activation of plant immunity is also often correlated with the upregulation of plant hormone regulatory miRNAs, as there is a trade-off between plant growth and defense activities [13]. In our results, during early *B*. *cinerea* infection, almost half the upregulated sRNAs were miRNAs in striking comparison to only 2% of the downregulated sRNAs. We found the upregulation of miR167, miR159 and miR319, which could be mediating an early response through auxin, abscisic acid (ABA) and jasmonic acid (JA) signaling pathways, respectively, in *A*. *thaliana* during *B*. *cinerea* infection. In agreement with the importance of these miRNAs in the biotic stress response, Zhang et al., (2011) reported that *A*. *thaliana* inoculated with an avirulent strain of the bacterial pathogen *Pseudomonas syringae* pv. *tomato* carrying the effector protein avrRpt2 showed an upregulation of miR159 and miR319 at 14 hpi [34]. They reported the concomitant downregulation of the respective target genes, thus suggesting the repression of the components involved in the ABA and JA signaling pathways and the subsequent enhancement of the salicylic acid (SA)-mediated defense. Furthermore, pointing out a role of these miRNAs in the interaction with *B*. *cinerea*. Jin and Wu (2015) and Wu et al., (2020) showed that sly-miR159 and sly-miR319 and their respective target genes are also upregulated and downregulated, respectively, in tomato during fungal infection [20,59]. They also reported that *A*. *thaliana* overexpressing miR319c showed not only the downregulation of the miR319c target gene TCP2, but also an increased resistance to *B*. *cinerea* infection, pointing out TCP2 as a negative regulator of *A*. *thaliana* resistance to *B*. *cinerea* infection. Taken together, these data indicate that expression regulation of miR159, miR319 and their target genes is important for activating appropriate defenses during an early stage of interaction between *A*. *thaliana* and *B*. *cinerea*.

### Known cross-kingdom *A*. *thaliana* miRNAs and TAS-derived secondary siRNAs as early responsive sRNAs to *B*. *cinerea*

Zhang et al., (2016) and Cai et al., (2018) reported that plant cells export sRNAs into fungal cells by means of extracellular vesicles, mediating cross-kingdom gene silencing of fungal genes, contributing to disease resistance [25,60]. Cai et al., (2018) showed that the TAS1c- and TAS2-derived secondary siRNAs TAS1c-siR483 and TAS2-siR453 are selectively loaded into plant extracellular vesicles to be transported to *B*. *cinerea* cells for the silencing of fungal virulence factor genes as part of the *A*. *thaliana* defense mechanisms [25]. They also detected TAS1c-siR483, TAS2-siR453, IGN-siR1 (hc-sRNA) and miR166 within *B*. *cinerea* protoplasts purified from *B*. *cinerea-*infected *A*. *thaliana*. Zhang et al., (2016) reported that cotton miRNAs miR166 and miR159 are also capable of cross-kingdom silencing of virulence-related genes, in this case, with the fungal pathogen *Verticillium dahliae* [60]. They showed that these two plant miRNAs were present inside the fungal cells and induced upon infection, while their putative fungal target genes, Ca^2+^-dependent cysteine protease (Clp-1) and isotrichodermin C-15 hydroxylase (HiC-15), were downregulated in fungal hyphae. We found that TAS1c-siR483, miR166e-3p/f/b-3p/a-3p/g/c/d/h/i and miR159b-3p were upregulated in response to *B*. *cinerea*, being part of the topmost abundantly expressed sRNAs (Fig 3). Taken together, these data suggest that the upregulation of vesicle-transported sRNAs TAS1c-siR483 and possibly miR166, and miR159 is important in early cross-kingdom gene silencing defense response.

### Early expression regulation of sRNA-mediated silencing related genes by sRNAs during *B*. *cinerea* infection

In this study, we explored GO term enrichment analysis for the putative target genes of the differentially expressed sRNAs. The GO term enrichment analysis revealed that target genes are involved in different biological processes. Notably, we found high enrichment for the GO term “primary miRNA processing (GO:0031053)”. One of the genes associated with this term was *DCL1*, which is regulated by miR162. Zhang et al., (2015) reported in rice the downregulation of *OsDCL1* and the upregulation of osa-miR162a when treated with the rice blast pathogen *Magnaporthe oryzae* [61]. They also reported that rice *OsDCL1* RNAi lines constitutively expressed defense related genes and were also more resistant to virulent strains of *M*. *oryzae*, hypothesizing a negative role for OsDCL1 in rice immunity. Here, we report that upregulation of miR162b-3p/a-3p and downregulation of *DCL1* occurred at an early stage of *B*. *cinerea* infection. Rajagopalan et al., (2006) reported that in addition to miR162, miR838 is another miRNA with the potential to regulate *DCL1* post-transcriptionally [62]. This miRNA was derived from a hairpin within the 14th intron of the *DCL1* pre-mRNA and it was proposed that the presence of this intronic miRNA enables a self-regulatory mechanism that helps maintaining DCL1 homeostasis in *A*. *thaliana*. We found the upregulation of miR838 with *B*. *cinerea* treatment (S9 Fig), and we also propose other possible targets for miR838 (S7 Table) for which it could be interesting to validate the post-transcriptional regulation during early *B*. *cinerea* infection. Additionally, we noticed there was another sRNA biogenesis related gene associated with an enriched GO term; in this case *AGO2* was associated with the “response to stimulus (GO:0050896)” term. Harvey et al., (2011) characterized *AGO2* as an antiviral defense gene that responds to several plant viruses [40]. They mentioned that AGO1 represents a first layer of RNA-mediated defense in the interactions between plants and viruses, which can be inactivated by some viruses that produce AGO1 silencing suppressors. They also mentioned that this therefore activates a second layer of RNA-mediated defense where *AGO2* mRNA is no longer suppressed by AGO1-associated miR403. In our results, miR403-3p was upregulated and *AGO2* was downregulated by *B*. *cinerea* infection. These results suggest that post-transcriptional regulation of *DCL1* and *AGO2* transcripts, and therefore of the sRNA-mediated silencing related genes, could be important for appropriate immune responses in *A*. *thaliana* at an early stage of interaction with *B*. *cinerea*.

### PPR gene targeting by miRNAs and TAS-derived secondary siRNAs as an early plant responsive mechanism to *B*. *cinerea*

Associated with the enriched GO term “biological regulation (GO:0065007)”, we found a *PPR* gene (AT5G55840) for which it has been previously validated to be regulated by miR161.2 (Ana Karen Ávila Sandoval, personal communication, June, 2023). Hou et al. (2019) found that miR161, which triggers the production of secondary siRNAs derived from a subset of *PPR* gene transcripts, contributed to *A*. *thaliana* defense against the oomycete pathogen *Phytophthora capsici* [31]. They reported that miR161 and the PPR-derived secondary siRNAs, PPR-siRNA-1 and PPR-siRNA-2 were upregulated upon challenge with *P*. *capsici*. They also reported that *A*. *thaliana* lines overexpressing *MIR*161 showed enhanced resistance to *P*. *capsici* while *MIR*161 knock-out lines were hypersusceptible. In addition, the *A*. *thaliana* mutant lines of *RDR6* and *SGS3* (enzymes involved in the secondary siRNA production) were also hypersusceptible to the pathogen, suggesting a role for the secondary siRNA pathway in plant defense during *P*. *capsici* infection. These PPR-derived secondary siRNAs were found in extracellular vesicles and likely silenced target genes in *P*. *capsici* during natural infection. In this sense, future work will allow us to better understand the regulation of miR161.2, miR400 and TAS2-siR165 on *PPR* genes (such as AT1G62914, AT5G41170, AT5G55840 and AT5G65560), which possibly participate in the production of PPR-derived secondary siRNAs that silence target genes in *B*. *cinerea*.

## Conclusions

This work reports the first transcriptome-wide catalogue of the responsive small RNAs to *B*. *cinerea* infection during the early interaction with *A*. *thaliana*. Different classes of *A*. *thaliana* sRNAs undergo changes in their accumulation profiles at this early stage of the interaction. Downregulated sRNAs mainly belong to the hc-siRNA class, suggesting an early role of these sRNAs in gene expression regulation through DNA methylation and chromatin remodeling. Remarkably, among the most accumulated sRNAs upon fungal infection we found that evolutionary conserved miRNAs were mainly upregulated. The question is to know if these sRNAs have an important role in other plant species and with different pathogens. Some of the corresponding mRNA targets are involved in various biological processes such as hormone response and production of secondary siRNAs derived from TAS1/2 genes and a subset of PPR genes. Furthermore, some of the differentially expressed sRNAs are known to be transported in extracellular vesicles into fungal cells likely silencing fungal virulence factors. It has been described that expressing responsive sRNAs in *A*. *thaliana* and tomato attenuates fungal pathogenicity, and thus the sRNAs catalogue described in this work contributes to further understand the sRNA-mediated defense mechanisms used by the plant against pathogens to design new strategies that can help stop the infection of this agronomically important pathogen.

## Supporting information

S1 Fig Principal component analysis for sRNA and mRNA read counts from mock and B. cinerea treated samples (6 hpi). There were two replicates for each treatment, read counts were normalized by *rlog transformation*. Plots for sRNAs and mRNAs are shown on left and right, respectively.
(TIFF)

S2 Fig Length distribution of reads mapped to A. thaliana genome. There were two replicates from mock and *B*. *cinerea* treatments.
(TIFF)

S3 Fig Saturation analysis. Reads corresponding to miRNA or secondary siRNA sequences were randomly subsampled from our cleaned libraries and the number of detected non-redundant sRNA sequences was evaluated as subsampled reads were added. We used 10 read counts as detection threshold. The graph shows the number of newly detected non-redundant sRNA sequences.
(TIFF)

S4 Fig Stem-loop RT-qPCR validation of miRNAs responsive to B. cinerea infection. The plot shows the expression profile of miR403-3p and miR167b/a-5p/c. Total RNA samples obtained from mock and *B*. *cinerea* treated leaves (6 hpi) were used to determine miRNA accumulation by RT‐qPCR. For each treatment, a total of fifteen leaves were used (five leaves from each of three plants). Amplification of miRNAs was normalized with U6 snRNA and relative expression was calculated using the formula 2^-ΔΔCt^. Error bars represent standard deviation of three technical replicates. Differential miRNA accumulation between mock and *B*. *cinerea* treatments is indicated by asterisks (p-value < 0.05 (*) and < 0.01 (**), as obtained from two-tailed independent samples t-tests).
(TIFF)

S5 Fig Upregulation of miRNAs involved in the fine-tuning of hormone signaling pathways. The plot shows the expression profile of miR167, miR159, miR319 and their targets between mock and *B*. *cinerea* treatments. Normalized counts of expression values were obtained using the DESeq2 algorithm. Error bars represent standard deviation of two biological replicates. Differential expression between mock and *B*. *cinerea* treatments is indicated by asterisks (adjusted p-value < 0.05 (*) and < 0.001 (***), or no significant (N.S.), as obtained from differential expression analysis using DESeq2). Arrows connect miRNAs with their target genes.
(TIFF)

S6 Fig Secondary siRNAs derived from the TAS1c transcript from AT2G39675 locus, biogenesis of which are triggered by miR173. miR173 mediates the cleavage of the TAS1c primary transcript, at the position indicated by the red arrow. This cleavage triggers the dsRNA conversion of the resulting downstream cleaved RNA fragment and the sequential production of the secondary siRNAs at approximated 21 nts phase intervals, starting at the miR173 cleavage site. Sequences in color indicate the secondary siRNAs that Cai et al. (2018) [25] described that are loaded in *A*. *thaliana* extracellular vesicles.
(TIFF)

S7 Fig Secondary siRNAs derived from the TAS2 transcript from AT2G39681 locus, biogenesis of which are triggered by miR173. miR173 mediates the cleavage of the TAS2 primary transcript, at the position indicated by the red arrow. This cleavage triggers the dsRNA conversion of the resulting downstream cleaved RNA fragment and the sequential production of the secondary siRNAs at an approximated 21 nts phase intervals, starting at the miR173 cleavage site. Sequences in color indicate the secondary siRNAs that Cai et al. (2018) [25] described that are loaded in *A*. *thaliana* extracellular vesicles.
(TIFF)

S8 Fig Differentially expressed mRNAs during B. cinerea infection. Differentially expressed mRNAs are indicated by orange dots and mRNAs without differential expression by gray dots (adjusted p-value ≤ 0.05, as obtained from differential expression analysis using DESeq2). The numbers at the corners indicate upregulated (above) or downregulated (below) mRNAs in *B*. *cinerea* treatment compared with mock.
(TIFF)

S9 Fig miR838 is upregulated during B. cinerea infection. The plot shows the expression profile of miR838 between mock and *B*. *cinerea* treatments. Normalized counts of expression values were obtained using the DESeq2 algorithm. Error bars represent standard deviation of two biological replicates. Differential expression between mock and *B*. *cinerea* treatment is indicated by an asterisk (adjusted p-value < 0.05 (*), as obtained from differential expression analysis using DESeq2).
(TIFF)

S1 Table Number of sequenced reads. (XLSX)

S2 Table Stem-loop RT-qPCR primer sequences. (XLSX)

S3 Table All expressed sRNAs from A. thaliana treated with mock or B. cinerea 6hpi. (XLSX)

S4 Table Differentially expressed sRNAs from A. thaliana treated with mock or B. cinerea 6hpi. (XLSX)

S5 Table All expressed mRNAs from A. thaliana treated with mock or B. cinerea 6hpi. (XLSX)

S6 Table Differentially expressed mRNAs from A. thaliana treated with mock or B. cinerea 6hpi. (XLSX)

S7 Table Integrated data of anticorrelated differentially expressed sRNAs and putative mRNA targets from A. thaliana treated with mock or B. cinerea 6hpi. (XLSX)

S8 Table Enriched GO terms and putative mRNA targets associated with them. (XLSX)
