# Supplementary figures and images for: Identification of Arabidopsis thaliana small RNAs responsive to the fungal pathogen Botrytis cinerea at an early stage of interaction
Source: PLoS One. 2024 Jun 14;19(6):e0304790. doi: 10.1371/journal.pone.0304790 (PMC11178217; doi:10.1371/journal.pone.0304790)

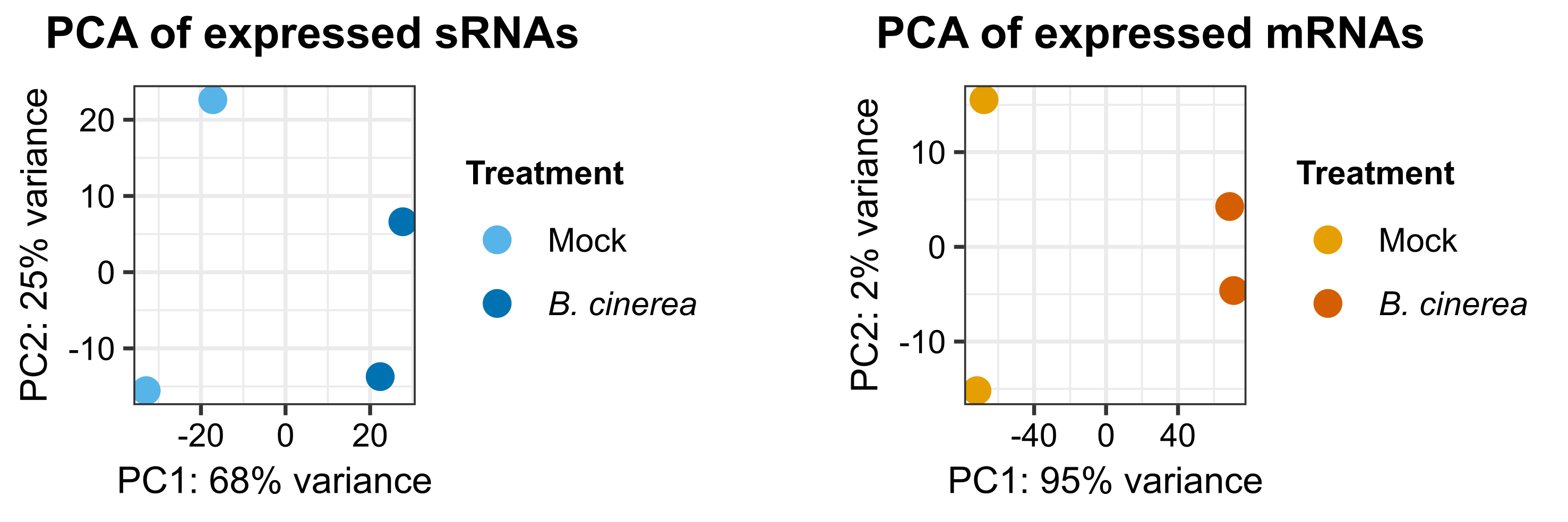

Supplement: S1 Fig — There were two replicates for each treatment, read counts were normalized by rlog transformation. Plots for sRNAs and mRNAs are shown on left and right, respectively. (TIFF) [file pone.0304790.s001.tiff]

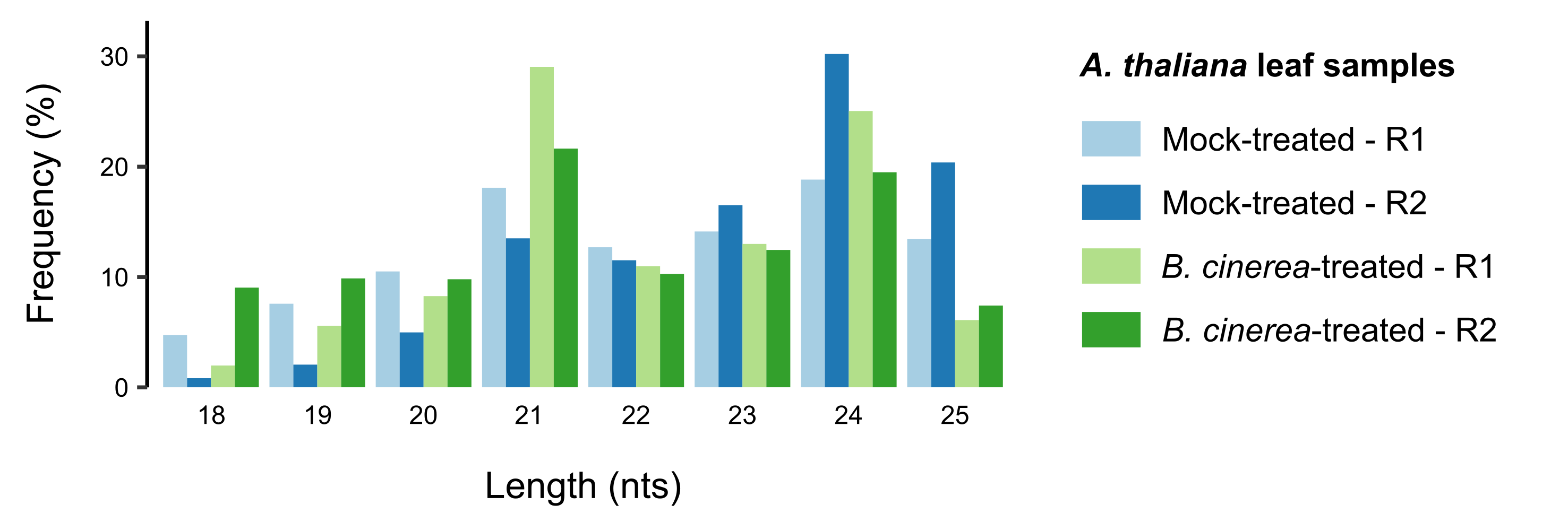

Supplement: S2 Fig — There were two replicates from mock and B. cinerea treatments. (TIFF) [file pone.0304790.s002.tiff]

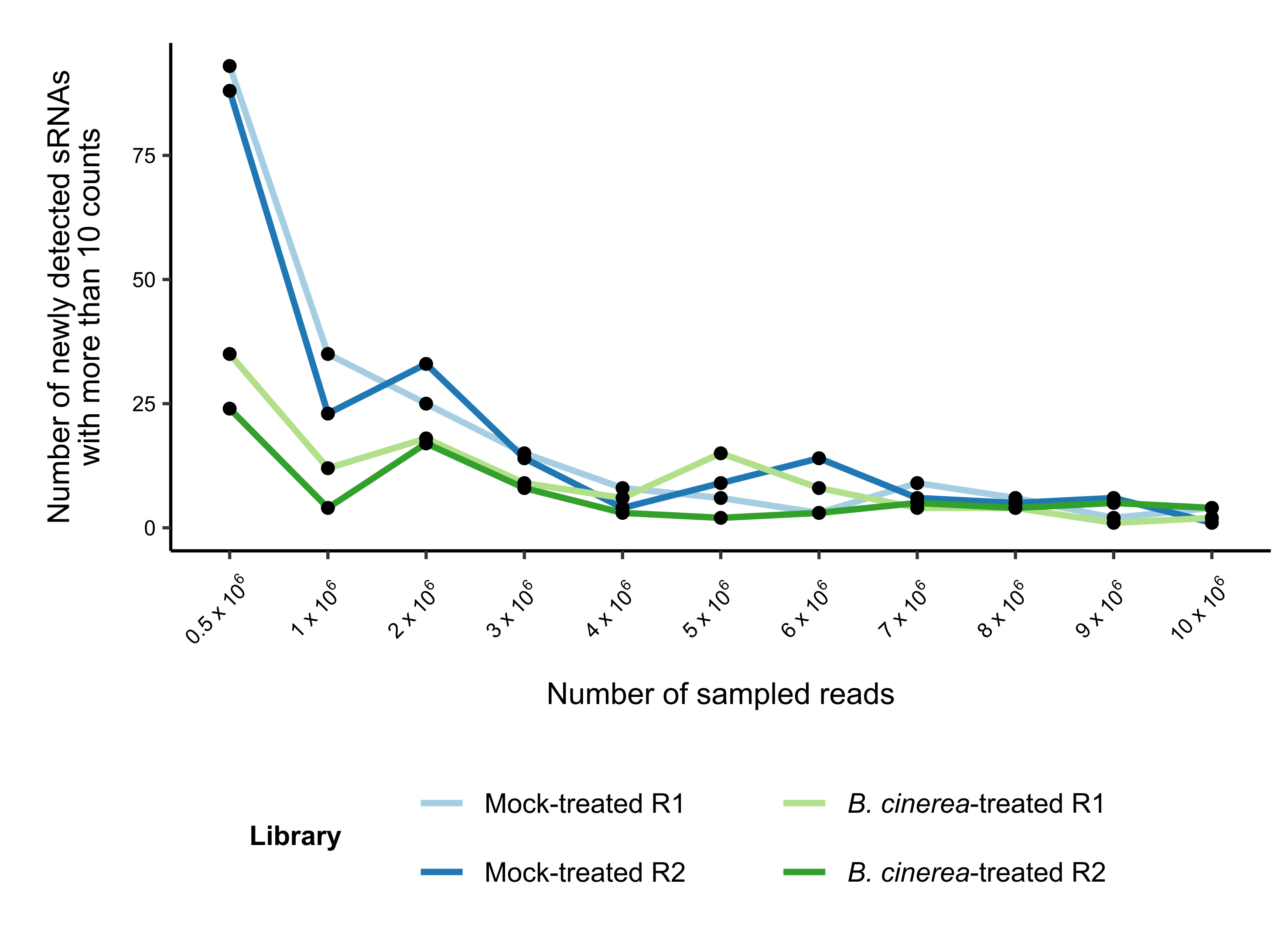

Supplement: S3 Fig — Reads corresponding to miRNA or secondary siRNA sequences were randomly subsampled from our cleaned libraries and the number of detected non-redundant sRNA sequences was evaluated as subsampled reads were added. We used 10 read counts as detection threshold. The graph shows the number of newly detected non-redundant sRNA sequences. (TIFF) [file pone.0304790.s003.tiff]

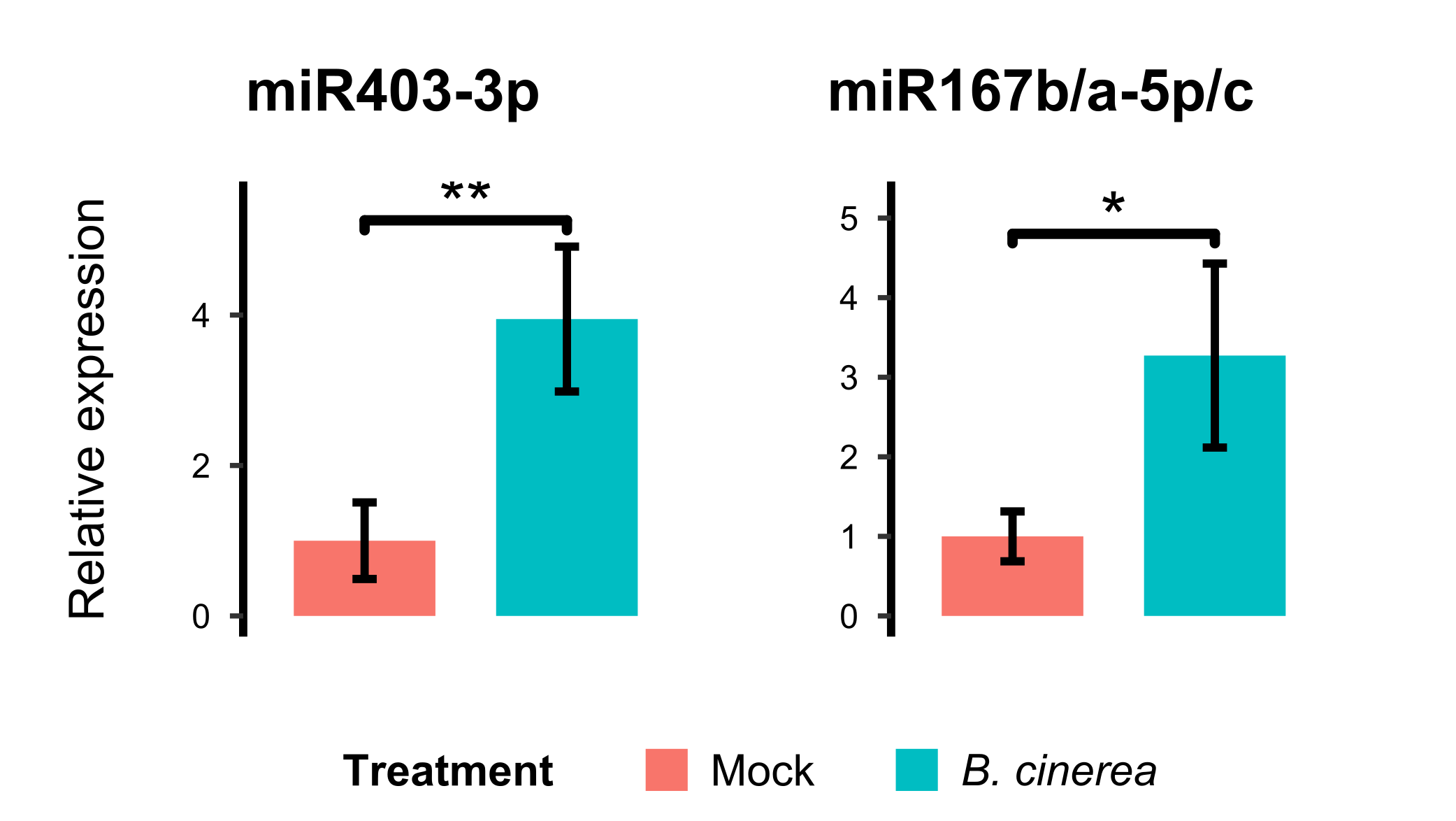

Supplement: S4 Fig — The plot shows the expression profile of miR403-3p and miR167b/a-5p/c. Total RNA samples obtained from mock and B. cinerea treated leaves (6 hpi) were used to determine miRNA accumulation by RT‐qPCR. For each treatment, a total of fifteen leaves were used (five leaves from each of three plants). Amplification of miRNAs was normalized with U6 snRNA and relative expression was calculated using the formula 2-ΔΔCt. Error bars represent standard deviation of three technical replicates. Differential miRNA accumulation between mock and B. cinerea treatments is indicated by asterisks (p-value < 0.05 (*) and < 0.01 (**), as obtained from two-tailed independent samples t-tests). (TIFF) [file pone.0304790.s004.tiff]

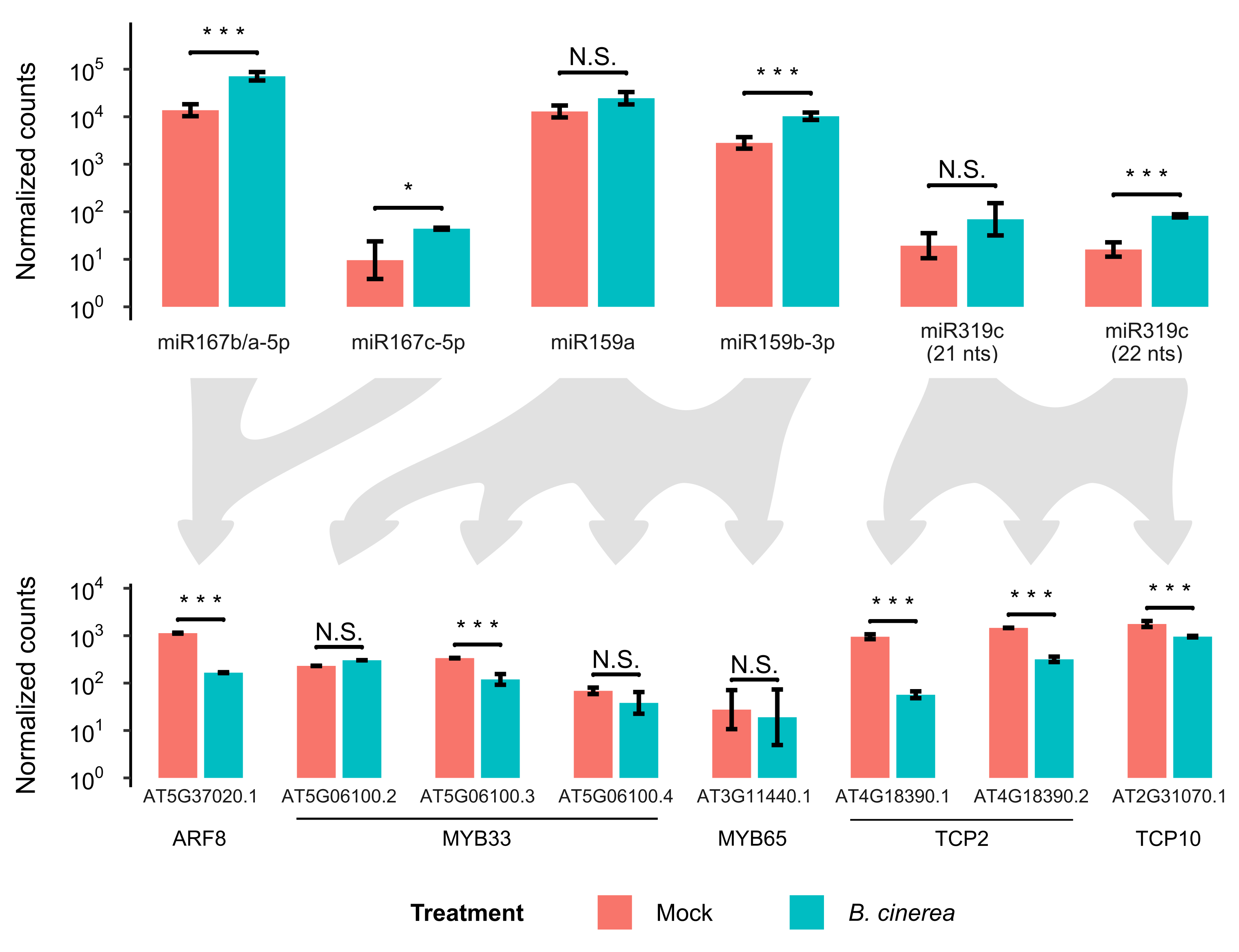

Supplement: S5 Fig — The plot shows the expression profile of miR167, miR159, miR319 and their targets between mock and B. cinerea treatments. Normalized counts of expression values were obtained using the DESeq2 algorithm. Error bars represent standard deviation of two biological replicates. Differential expression between mock and B. cinerea treatments is indicated by asterisks (adjusted p-value < 0.05 (*) and < 0.001 (***), or no significant (N.S.), as obtained from differential expression analysis using DESeq2). Arrows connect miRNAs with their target genes. (TIFF) [file pone.0304790.s005.tiff]

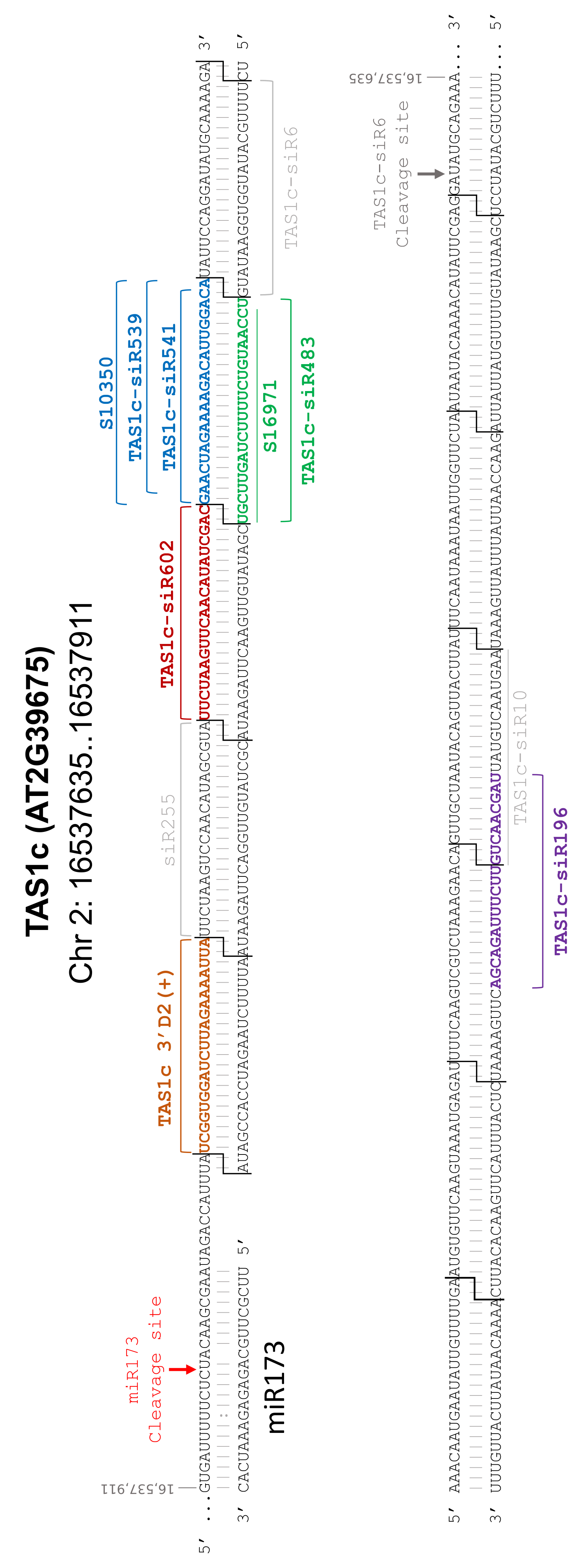

Supplement: S6 Fig — miR173 mediates the cleavage of the TAS1c primary transcript, at the position indicated by the red arrow. This cleavage triggers the dsRNA conversion of the resulting downstream cleaved RNA fragment and the sequential production of the secondary siRNAs at approximated 21 nts phase intervals, starting at the miR173 cleavage site. Sequences in color indicate the secondary siRNAs that Cai et al. (2018) [25] described that are loaded in A. thaliana extracellular vesicles. (TIFF) [file pone.0304790.s006.tiff]

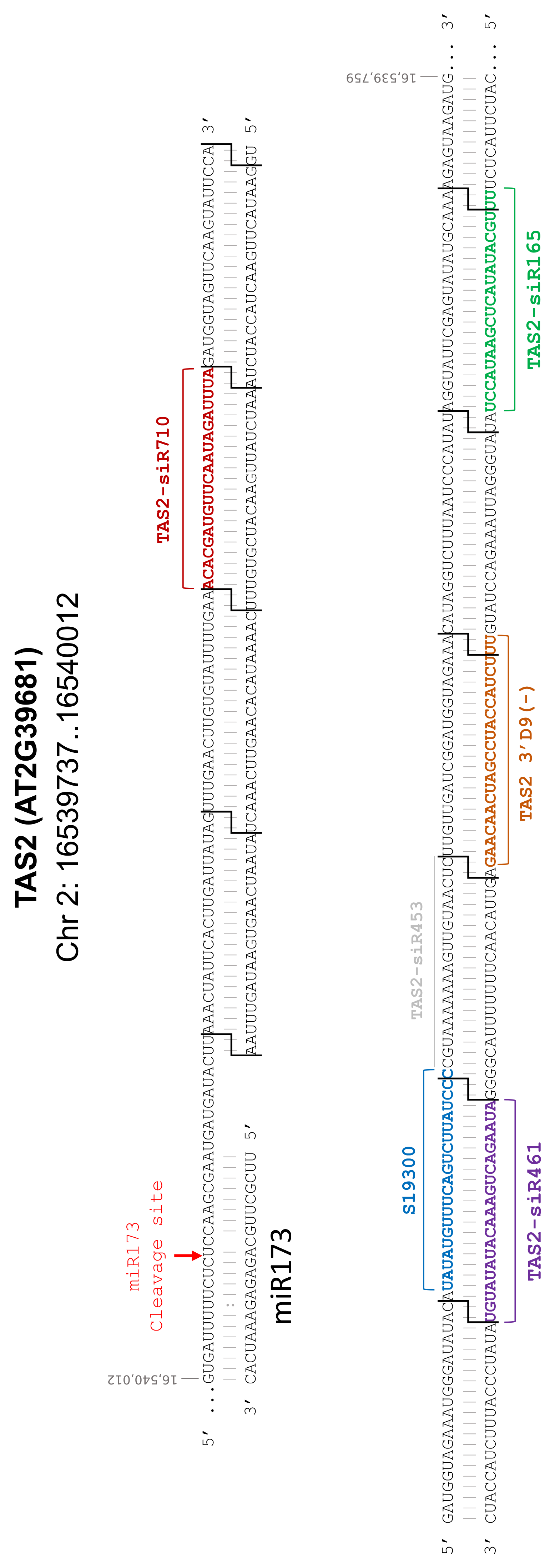

Supplement: S7 Fig — miR173 mediates the cleavage of the TAS2 primary transcript, at the position indicated by the red arrow. This cleavage triggers the dsRNA conversion of the resulting downstream cleaved RNA fragment and the sequential production of the secondary siRNAs at an approximated 21 nts phase intervals, starting at the miR173 cleavage site. Sequences in color indicate the secondary siRNAs that Cai et al. (2018) [25] described that are loaded in A. thaliana extracellular vesicles. (TIFF) [file pone.0304790.s007.tiff]

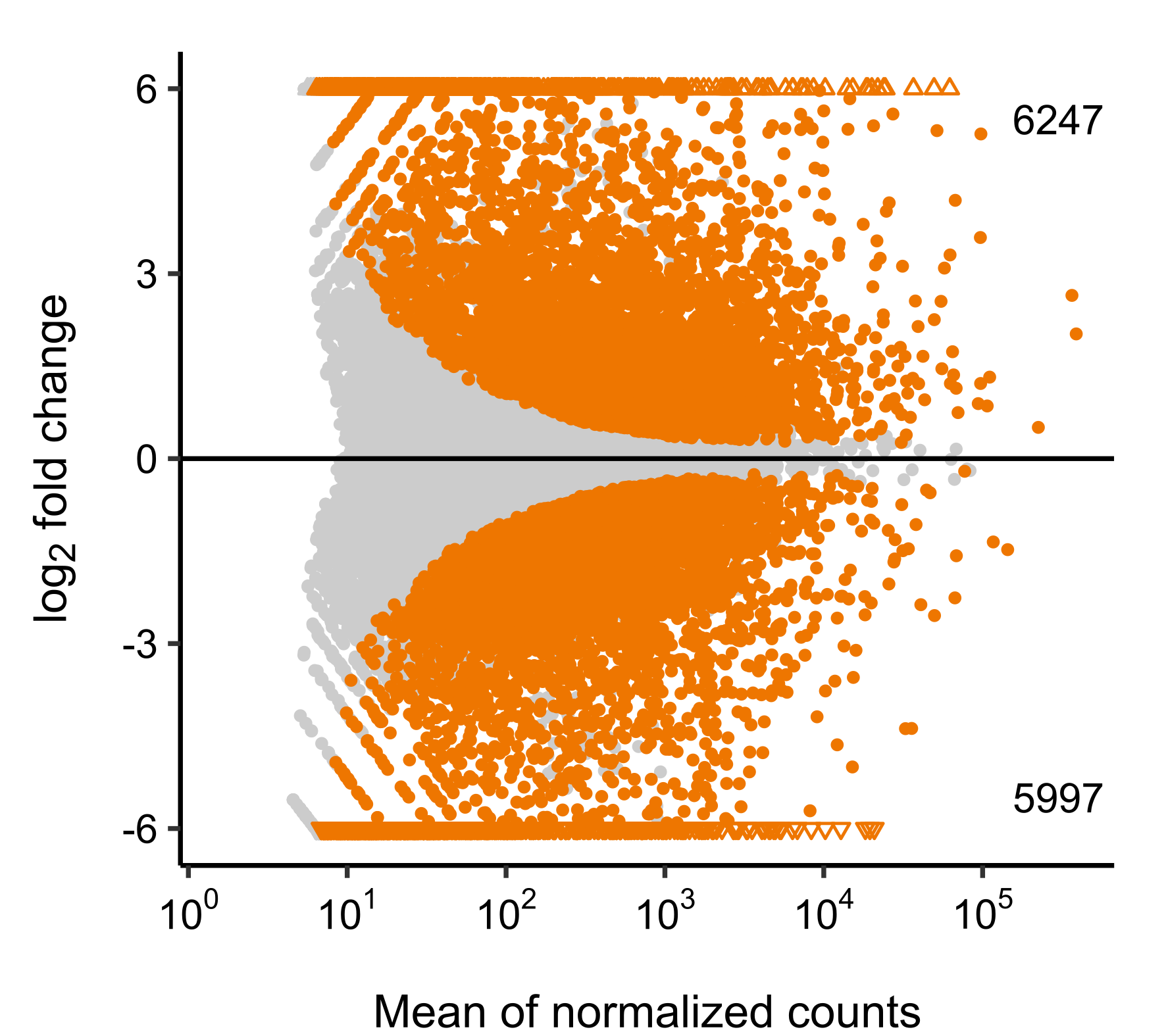

Supplement: S8 Fig — Differentially expressed mRNAs are indicated by orange dots and mRNAs without differential expression by gray dots (adjusted p-value ≤ 0.05, as obtained from differential expression analysis using DESeq2). The numbers at the corners indicate upregulated (above) or downregulated (below) mRNAs in B. cinerea treatment compared with mock. (TIFF) [file pone.0304790.s008.tiff]

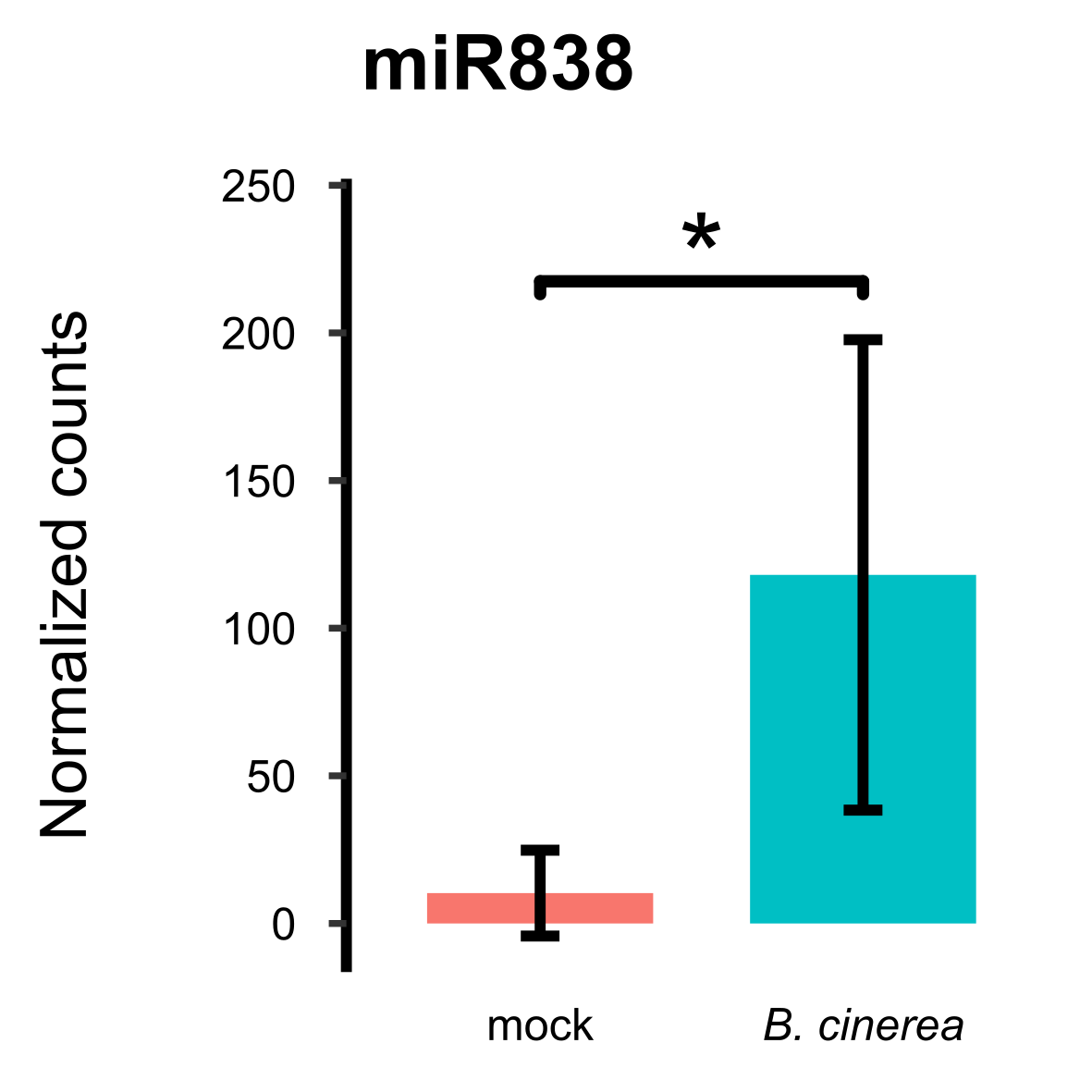

Supplement: S9 Fig — The plot shows the expression profile of miR838 between mock and B. cinerea treatments. Normalized counts of expression values were obtained using the DESeq2 algorithm. Error bars represent standard deviation of two biological replicates. Differential expression between mock and B. cinerea treatment is indicated by an asterisk (adjusted p-value < 0.05 (*), as obtained from differential expression analysis using DESeq2). (TIFF) [file pone.0304790.s009.tiff]
